# Supplementary material for: Comparison of 3 optimized delivery strategies for completion of isoniazid-rifapentine (3HP) for tuberculosis prevention among people living with HIV in Uganda: A single-center randomized trial
Source: PLoS Med. 2024 Feb 20;21(2):e1004356. doi: 10.1371/journal.pmed.1004356 (PMC10914279; doi:10.1371/journal.pmed.1004356)
Supplement: S1 Text — (DOCX) [file pmed.1004356.s001.docx]

**Supplemental Methods.** Trial inclusion and exclusion criteria.

**Inclusion criteria:**

1. No suspicion of active tuberculosis based on World Health Organization (WHO) symptom screen or elevated point-of-care C-reactive protein test
2. Not currently taking or planning to start treatment for active TB
3. Not previously completed treatment for active TB or at least 6 months of isoniazid (INH) preventive treatment with latent TB within the past 2 years
4. No known sensitivity/intolerance/resistance to INH or rifamycins
5. No known contact with a person with INH- or rifamycin-resistant TB
6. not taking antiretroviral medications or other medications contraindicated for use with rifapentine (e.g., warfarin, phenytoin)
7. Not pregnant, breast feeding or intending to get pregnant in the next 120 days

**Exclusion criteria:**

1. Weight <40 kilograms^*^
2. New on ART (initiated ART within the previous 3 months)
3. Pre-existing documentation of clinical liver disease
4. History of alcohol abuse
5. Prisoners
6. Measured baseline serum alanine aminotransferase or aspartate aminotransferase level more than 3 times the upper limit of normal
7. Those not intending to stay within 25 kilometers of the study clinic or receive further care at the study clinic (to enable proper follow-up)
8. No access to a mobile telephone or not willing to receive phone call reminders (which would interfere with the SAT delivery strategy)
9. Those living with another household member already enrolled in the study

*Similar to previous implementation trials of 3HP (twelve weeks of once-weekly isoniazid and rifapentine), participants were required to weigh 40 kilograms or more to be eligible for trial participation, as medication dosage was not adjusted for participant weight. Weighing less than 40 kilograms resulted in one participant being excluded from trial participation.
