# Supplementary material for: Comparison of 3 optimized delivery strategies for completion of isoniazid-rifapentine (3HP) for tuberculosis prevention among people living with HIV in Uganda: A single-center randomized trial
Source: PLoS Med. 2024 Feb 20;21(2):e1004356. doi: 10.1371/journal.pmed.1004356 (PMC10914279; doi:10.1371/journal.pmed.1004356)
Supplement: S2 Text — (PDF) [file pmed.1004356.s002.pdf]

Statistical Analysis Plan: 3HP Options Trial

OFFICIAL

<table>
<tr><td>Full Title</td><td colspan="3">Options for Delivering Isoniazid-Rifapentine (3HP) for TB Prevention (3HP Options Implementation Trial)</td></tr>
<tr><td>Acronym</td><td colspan="3">3HP Options Trial</td></tr>
<tr><td rowspan="6">Document History</td><td>Version No.</td><td>Version Date</td><td>Description of Change</td></tr>
<tr><td>1.0</td><td>2021-03-31</td><td>Initial release</td></tr>
<tr><td>1.1</td><td>2022-04-26</td><td>1. Change of primary outcome timeline from 16 weeks from date of enrolment to 16 weeks from date of treatment initiation date.
2. Clarification that the per protocol analysis is the equivalent of the secondary outcome of the proportion who complete treatment among those who initiate.
3. Addition of history of prior tuberculosis (TB) as a pre-specified subgroup analysis.</td></tr>
<tr><td></td><td></td><td></td></tr>
<tr><td></td><td></td><td></td></tr>
<tr><td></td><td></td><td></td></tr>
<tr><td>Trial Registration</td><td colspan="3">Clinicaltrials.gov: NCT03934931</td></tr>
<tr><td>Principal Investigators</td><td colspan="3">Adithya Cattamanchi, David Dowdy, Fred Semitala</td></tr>
</table>

Signature Page

The signature below constitutes the approval of this statistical analysis plan and the attachments and provides the necessary assurances that this trial analysis will be conducted according to all stipulations of the plan.

Uganda Study Site Principal Investigator:

DocuSigned by:
Fred Semitala
0024A685AE784E8...

Signed: ______________________________ Date: ___4/26/2022__________
Fred C. Semitala MB.Ch.B, M.Med, MPH, ImS

Overall Study Principal Investigators:

DocuSigned by:
Adithya Cattamanchi
5E4BA1E695CB406...

Signed: ______________________________ Date: ___4/26/2022__________
Adithya Cattamanchi, MD, MAS

DocuSigned by:
David Dowdy
F4298EFA7B59448...

Signed: ______________________________ Date: ___4/26/2022__________
David W. Dowdy, PhD

Trial Statisticians:

DocuSigned by:
Noah Kiwanuka
DAF61C80B244479...

Signed: ______________________________ Date: ___4/26/2022__________
Noah Kiwanuka, PhD

DocuSigned by:
Patrick Phillips
F39BACE611F44E6...

Signed: ______________________________ Date: ___4/26/2022__________
Patrick PJ Phillips, PhD

1. Introduction

1.1 Aim

To compare the uptake of a 3 month (12 dose) regimen of isoniazid and rifapentine (**3HP**) under three delivery strategies: Directly Observed Therapy (**DOT**); Self-administered Therapy (**SAT**); or Patient choice (with the help of a decision aid) of either DOT or SAT among people living with HIV (**PLHIV**) accessing HIV/AIDS care at Mulago Immune Suppression Syndrome (**ISS**; i.e. HIV/AIDS) clinic in Kampala, Uganda.

2. Background and Objectives

2.1 Specific Objectives

Primary Objective: To compare the uptake of 3HP under three delivery strategies: 1) Facilitated DOT; 2) Facilitated SAT; and 3) Informed patient choice (using a decision aid) between facilitated DOT and facilitated SAT. The primary outcome will be defined as the proportion of eligible participants who accept treatment and take at least 11 of 12 doses of rifapentine (**RPT**)/isoniazid (**INH**) within 16 weeks of treatment initiation.

Secondary Objectives:

1. To estimate the costs and compare the cost-effectiveness of three strategies for delivering 3HP.
2. To identify processes and contextual factors that influence patient acceptance and completion of 3HP under each delivery strategy.
3. To identify clinic-level barriers to adoption and implementation of 3HP under each delivery strategy.
4. To determine the proportion of patients for whom 3HP treatment is discontinued due to adverse events (**AEs**)/intolerance.
5. To determine the cumulative 16-month incidence of active tuberculosis (**TB**) in each arm.

2.2 Primary hypotheses:

1. The proportion who accept and complete 3HP will be highest among PLHIV randomized to the informed choice arm.
2. The proportion of PLHIV who accept and complete 3HP can exceed 80% in a high HIV/TB burden setting.

2.3 General comments

This statistical analysis plan covers all effectiveness, implementation, and cost-effectiveness/modelling outcomes. Trial results reporting will follow the CONSORT 2010 statement extension for randomized control trials.[1]

This document summarizes effectiveness outcomes analysis for the primary outcome of the proportion of eligible participants who accept treatment and take at least 11 of 12 doses of RPT/INH within 16 weeks of treatment initiation and secondary outcomes for the implementation and cost-effectiveness of each delivery strategy. These endpoints will be evaluated through review of study documents and study registers for all patients enrolled in the 3HP Options Trial at Mulago ISS Clinic. This Statistical Analysis Plan is written in support of and is predominantly consistent with the full trial protocol version 1.4 (3HP_Protocol_Official_v1.4_12MAY2022); however, this analysis plan takes precedence.

2.4 Outcomes

The primary outcome assessing the effectiveness of delivery options is the proportion of patients accepting and completing 3HP treatment. Secondary outcomes are classified within RE-AIM domains and are described in full in the trial protocol.

Primary Outcome

Outcome	Numerator	Denominator
Proportion accepting and completing 3HP treatment	Number who take at least 11 of 12 doses within 16 weeks from date of treatment initiation	Number randomized

2.5 Sample Size

We estimated our sample size for the primary outcome (N=1656; 552/arm) based on a minimum clinically important difference of 10% in 3HP completion, comparing patient choice vs. DOT arms. Of note, the Centers for Disease Control and Prevention (**CDC**) TBTC iAdhere study of 3HP delivery strategies chose a 15% non-inferiority margin between DOT and SAT based on cost-effectiveness modeling in the US.[2] We chose 10% to be more conservative because similar modeling studies have not been done in low-income settings, and because ours is not a non-inferiority design. To be maximally conservative, we applied a Bonferroni correction based on two independent comparisons (choice vs. DOT and choice vs. SAT). Assuming a two-sided alpha of 0.025, and 5% loss between consent and allocation, a sample size of 552 participants per arm (N=1656 total) is required to provide power of 0.90 to detect this difference (Table 3). This sample size will also give us power of 0.85 to detect a point estimate of at least 80% effectiveness in the patient choice arm, assuming a true effectiveness of 85%. If the true effectiveness rises to 86%, our power to show effectiveness >80% increases to 0.96. Power was calculated using the PS - Power and Sample Size program.[3]

Power to detect 10% difference

Effectiveness		
**DOT**	**Choice**	**Power**
70%	80%	0.90
75%	85%	0.94
80%	90%	0.98
85%	95%	0.998

3. Statistical Methods

3.1 General analysis principles

All primary analyses will involve assessment of all listed primary (effectiveness of delivery options and secondary (reach, effectiveness, adoption, and implementation) outcomes. Intent-to-treat **(ITT)** is a strategy for analyzing data that compares patients in the groups to which they were originally randomly assigned, irrespective of the treatment actually received. The ITT analysis will be the main statistical approach for determining intervention effectiveness. Per-protocol **(PP)** analyses reflect the inclusion in the analysis of patients who strictly adhered to the trial protocol. Based on these general analysis principles, the specific analyses to be conducted are described below.

3.2 Dataset assembly, cleaning and creation of new variables

Datasets will be extracted from REDCap, transferred to Stata statistical software, and re-arranged into working analytic files. At first, long format shall be used, but after cleaning and generation of relevant variables, data will be reshaped into a wide format for final analyses. Working analytic datasets will be checked, each variable at a time, for completeness, logical range, and consistency accuracy. Patient adherence data, for both directly observed and digitally confirmed doses, are stored in Everwell 99DOTS adherence database. Dosing reports extracted from the Everwell database containing detailed information on patient adherence, including date and time stamps associated with each dose taken, will be merged with trial data in order to summarize patient treatment adherence and overall treatment completion statistics.

3.3 Baseline assessment of patient characteristics

Baseline demographic and clinical characteristics will be assessed for all patients across arms in order to assess achievement of balance across arms by randomization. Characteristics may include but are not limited to: age, sex, prior TB, multidimensional poverty index (**MPI**), ART status, and years on ART.

3.4 Primary analysis of primary outcome (intervention effect analysis)

Primary outcome analysis will include the comparing the proportion of those completing at least 11 of 12 doses within 16 weeks from date of treatment initiation for those in the DOT vs. SAT vs. choice arms. This will involve:

1. **Unadjusted intent-to-treat comparisons of effectiveness between arms**: Participants randomized to the choice arm will be treated separately from those randomized to either facilitated DOT or facilitated SAT; the proportion of those in the patient choice arm who choose DOT or SAT will be measured and reported, but the primary outcome will consider all individuals in this arm as randomized to patient choice. The primary outcome using ITT comparisons will be the unadjusted risk ratio and 97.5% two-sided confidence intervals (**CIs**), and Fisher's exact test to determine statistical significance (two-sided alpha of 0.025, including Bonferroni correction).

2. **Simple calculation (using the exact binomial CIs) of the effectiveness of each delivery option, with a target of 80%.** Whether 80% acceptance and completion is achieved will be based on the lower bound of the Bonferroni-corrected 97.5% CI exceeding 0.80 in any of the three arms. We will reject our null hypothesis if the lower bound of the 97.5% CI does not exceed 80%.

3.5 Sensitivity analyses of primary outcome

1. **Assessing our primary outcome using multivariable log-binomial models that adjust for unbalanced characteristics or pre-specified characteristics measured in the baseline survey** (*e.g.,* ART status). We will compare categorical variables using Chi-square tests and determine between which arms the variable in question is unbalanced using pairwise comparisons. We will compare balance of continuous variables using ANOVA. If multivariable log-binomial models fail to converge, we will use a modified Poisson regression with robust estimates of variance. Comparison will be made to logistic regression models as well if the proportion of the outcome (those who accept and complete treatment) in our population is rare (≤15%). Log binomial regression will be used for secondary (multivariable adjusted) analyses. If multivariable log-binomial models fail to converge, we will use a modified Poisson regression with robust estimates of variance. Participants who decline to initiate 3HP after randomization will be counted as not accepting/completing treatment in the primary (ITT) analysis and will be followed over time for clinical outcomes.

   Analyses will be done in Stata using *glm, family(binomial) link(log)* command for log binomial models, or *glm, family(poisson) link(log) vce(robust)* command for modified Poisson models. The log binomial regression model will take the general form of:

   $log(p)= \beta_0 + \beta_1 X + e,$ ……………………………………………………………. *Equation 1*

2. **Assessing our primary outcome to estimate a per protocol estimate that reflects the difference in treatment completion rates in patients that actually initiate treatment**. Thus, participants who were randomized but declined to initiate treatment will be excluded from this analysis. Note: the per protocol analysis of the primary outcome will be equivalent to our assessment of the secondary outcome specified in the trial protocol of treatment completion (the proportion of those who complete at least 11 of 12 doses within 16 weeks of treatment initiation among those who take at least one dose of 3HP). Assessment of the per protocol outcome will involve 1) unadjusted comparisons of risk ratios and 97.5% two-sided CIs of effectiveness between arms, 2) simple calculation (using the exact binomial CIs) of the

effectiveness of each delivery option, with a target of >80%, and 3) comparisons of risk ratios and 97.5% two-sided CIs of effectiveness between arms, adjusted for unbalanced characteristics or pre-specified characteristics measured in the baseline survey. We will assess for differences in the ITT and PP analysis populations by comparing baseline characteristics such as age, sex, antiretroviral therapy (**ART**) status, time on ART and prior TB.

3. **Assessing our primary outcome to estimate an as treated estimate that reflects the difference in treatment completion rates in patients** classifying participants based on which delivery strategy they actually received, irrespective of which arm they were allocated to. This will involve 1) allocating choice participants to either DOT or SAT arm, 2) making unadjusted comparisons of risk ratios and 97.5% two-sided CIs of effectiveness between arms, 3) simple calculation (using the exact binomial CIs) of the effectiveness of each delivery option, with a target of 80% and 4) comparisons of risk ratios and 97.5% two-sided CIs of effectiveness between arms, adjusted for unbalanced characteristics or pre-specified characteristics measured in the baseline survey.

3.6 Sub-group analyses

1. Sub-groups will be analyzed for primary analyses, secondary analyses and sensitivity analyses. We will conduct exploratory sub-group analyses including by:
   a. **Sex**: Participants will be divided into groups by sex (male vs. female)
   b. **Age:** Participants will be divided into age categories (e.g. younger vs. older)
   c. **ART status:** Participants will be divided into groups by ART status (on ART vs. not on ART)
   d. **Time on ART:** Participants will be categorized into groups by length of time on ART (e.g. newly initiated on ART vs. ART experienced patients)
   e. **History of prior TB:** Participants will be categorized into groups by history of prior TB (prior TB vs. no prior TB)
   f. **Level of reimbursement:** Reimbursement for clinic-related visits was adjusted throughout the course of the trial in response COVID-related travel increases and Uganda Institutional Review Board (**IRB**) minimum reimbursement requirements (levels: 15,000 Ugandan Shillings (**USh**), 20,000 USh, 30,000 USh)

3.7 Analysis of secondary outcomes

Secondary outcome analysis will include:

1. **Reach outcome(s):** 1) Unadjusted comparisons across arms, and 2) simple calculation of the proportion of patients randomized who accept treatment in each arm.
2. **Effectiveness outcomes:** 1) Occurrence of AEs across arms will be assessed using Chi-square tests and logistic regression; 2) Incidence of active TB will be assessed using Kaplan-Meier curves, log-rank tests, and Cox proportional hazards methods as appropriate. Assessment for active TB occurs at 15-16 months post enrolment. Survival analyses will include:
   - An assessment of the incidence rate of TB, comparing across arms (DOT vs. SAT vs. Choice) using Kaplan Meier, time-to-event analyses. We will estimate the difference in the Kaplan Meier and Cox proportional hazard estimators for the survivor functions, comparing the arms to get hazard ratios and 95% CIs.
   - An assessment of the incidence rate per arm by drug compliance, which will include for each arm: a comparison of the incidence of active TB in those within the arm that complete at least 11 of the 12 doses within the allotted 16 weeks vs. the incidence of active TB in those within the arm that complete ≤10 doses of 3HP. We will estimate the difference in the Kaplan Meier and Cox proportional hazard estimators for the survivor functions, comparing the arms to get hazard ratios and 95% CIs.

3. **Adoption outcomes:** For each process metric, we will report the monthly proportion (and 95% CI) number, or median (interquartile range (**IQR**)) to assess the implementation of intervention components in each arm.
4. **Implementation outcomes:** Quantitative data will be assessed using multivariable regression models to compare by study arm patient survey data including a) patient costs; b) scores on questions related to patient barriers to TB preventative care; and c) patient satisfaction. Qualitative data will be analysed using appropriate software where open coding will be applied to generate codes and themes. Thematic groupings will be developed and reviewed to identify emergent themes within each domain of the coding framework and quotes that best represent each domain. Thematic interpretation will include collaborative development of a coding framework and detailed coding of transcripts.
5. **Health economics outcomes:** Outcomes include the 1) unit cost per patient accepting and completing 3HP under each delivery strategy (cost analysis) and 2) the incremental cost per disability adjusted life year (**DALY**) averted (cost-utility analysis). The cost analysis will estimate and compare the per-patient cost of delivering 3HP under each strategy and; 2) the cost-utility analysis will identify the economically preferred option if there is a difference in effectiveness between arms (and the more effective option is also the more expensive one). To identify the preferred option, we will assume a willingness to pay of $500 per DALY averted (as being comparable to other published studies) in the primary analysis. We will estimate the incremental cost per DALY averted of patient choice relative to facilitated DOT or SAT.

3.8 Interim analysis

1. **Background:** The decision to conduct an interim analysis of trial data and the type of analysis was determined by the Trial Steering Committee (**TSC**), comprised of 3 members not involved in the implementation of the trial nor on the investigative team, and without the Principal Investigators seeing outcome data disaggregated by study arm
2. **Statistical Analysis:** Determination of the timing and the contents of the interim analysis was made in consultation with the study statistician and with input from the TSC. We will calculate and report the proportion (and 95% CI) of patients accepting and completing 3HP treatment within 16 weeks of randomization, aggregated across all study arms. Selected secondary outcomes may also be reported and include treatment acceptance and occurrence of severe adverse events resulting in stopping of 3HP treatment. We will assess outcomes stratified by sex, age, and time on ART. Secondarily, we will take a Bayesian inference approach to estimate the posterior probability that the proportion of patients who accept and complete 3HP, denoted here as θ, exceeds 80% in at least one of the three study arms. We will use a Beta distribution to the outcome parameter θ and a non-informative flat prior, Beta(1,1), making it such that the prior has little influence on the posterior distribution. We will calculate the posterior probability that θ exceeds 0.8 by calculating the size of the tail of the posterior distribution that is above 0.8.

References

1. Campbell MK, Piaggio G, Elbourne DR, Altman DG, Group C. Consort 2010 statement: extension to cluster randomised trials. *BMJ.* 2012;345:e5661.
2. Belknap R, Holland D, Feng PJ, et al. Self-administered Versus Directly Observed Once-Weekly Isoniazid and Rifapentine Treatment of Latent Tuberculosis Infection: A Randomized Trial. *Ann Intern Med.* 2017;167(10):689-697.
3. Dupont WD, WD P. Power and Sample Size Calculations: A Review and Computer Program. *Controlled Clinical Trials.* 1990;11:116-128.
