# Supplementary material for: Comparison of 3 optimized delivery strategies for completion of isoniazid-rifapentine (3HP) for tuberculosis prevention among people living with HIV in Uganda: A single-center randomized trial
Source: PLoS Med. 2024 Feb 20;21(2):e1004356. doi: 10.1371/journal.pmed.1004356 (PMC10914279; doi:10.1371/journal.pmed.1004356)

**Supplement Figure 1.** Example of participant 99DOTS-based adherence calendars, including a participant on directly observed therapy (DOT) (top) and a participant on self-administered therapy (SAT) (bottom). The blue squares indicate treatment start/end dates, dark red squares indicate confirmed missed doses, light red squares indicate unconfirmed missed doses, light green squares indicate manually reported doses (doses 1-12 for DOT participants; doses 1, 6 and 12 for SAT participants), dark green squares indicate doses reported via toll-free phone call on the expected dose date (SAT only), orange squares indicate doses reported via toll-free phone call not on the expected dose date but within the allotted time window to count towards adherence (SAT only), and yellow squares indicate doses recorded via toll-free phone call that were reported using a phone number shared by another participant in the study (SAT only).

**
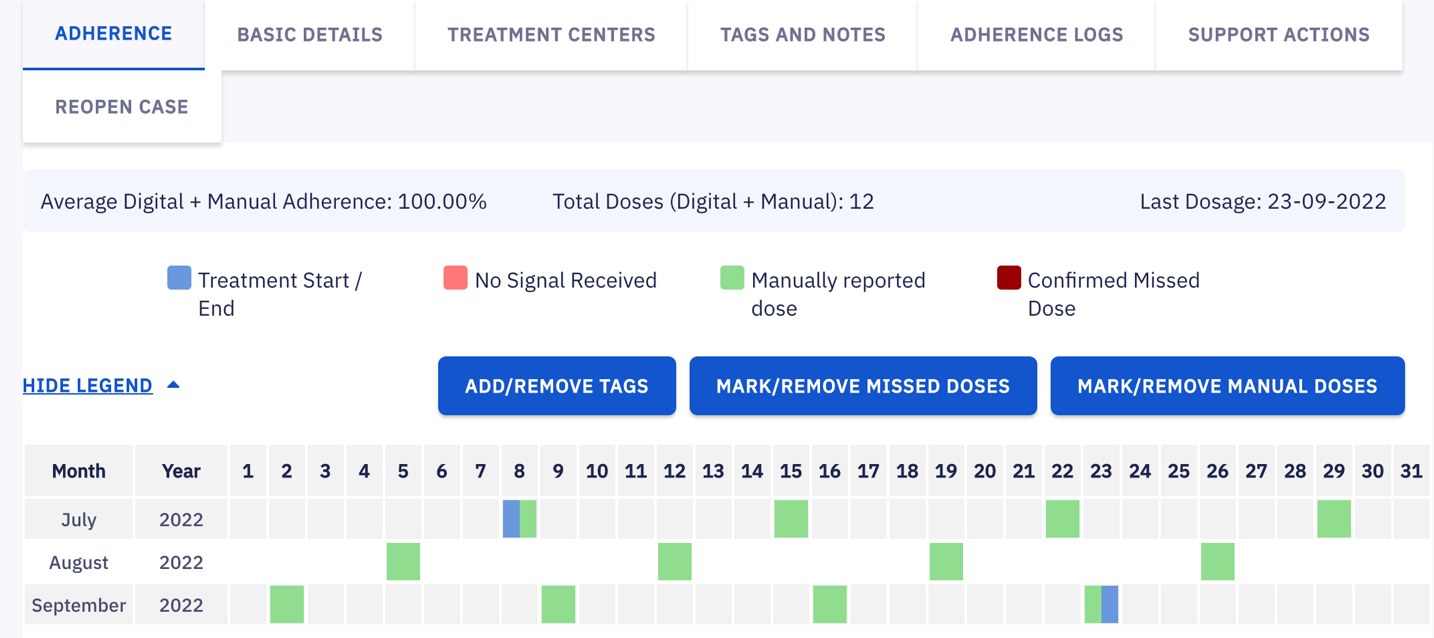
**


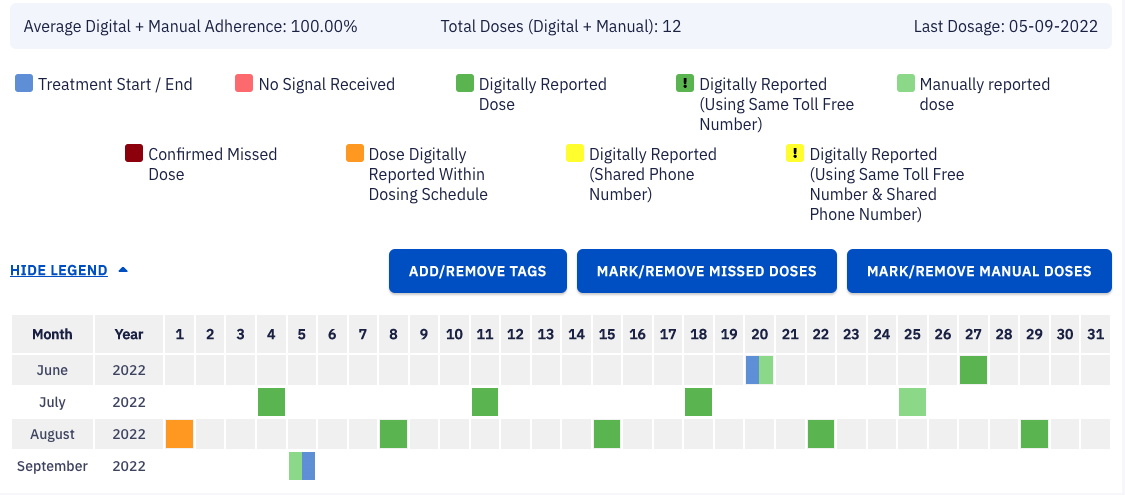

Supplement: S1 Fig — (DOCX) [file pmed.1004356.s003.docx]
