# Supplementary material for: Comparison of 3 optimized delivery strategies for completion of isoniazid-rifapentine (3HP) for tuberculosis prevention among people living with HIV in Uganda: A single-center randomized trial
Source: PLoS Med. 2024 Feb 20;21(2):e1004356. doi: 10.1371/journal.pmed.1004356 (PMC10914279; doi:10.1371/journal.pmed.1004356)

**Supplement Figure 2.** Pill packs were pre-sorted with plastic bags containing weekly 3HP doses (including pyridoxine pills) and weekly card inserts including a toll-free number to call to confirm dosing on the front, and a motivational message on the back. Pill packs also included an informational card with clinic contact information and dosing information that was filled by a routine clinical provider upon initiation of 3HP treatment.


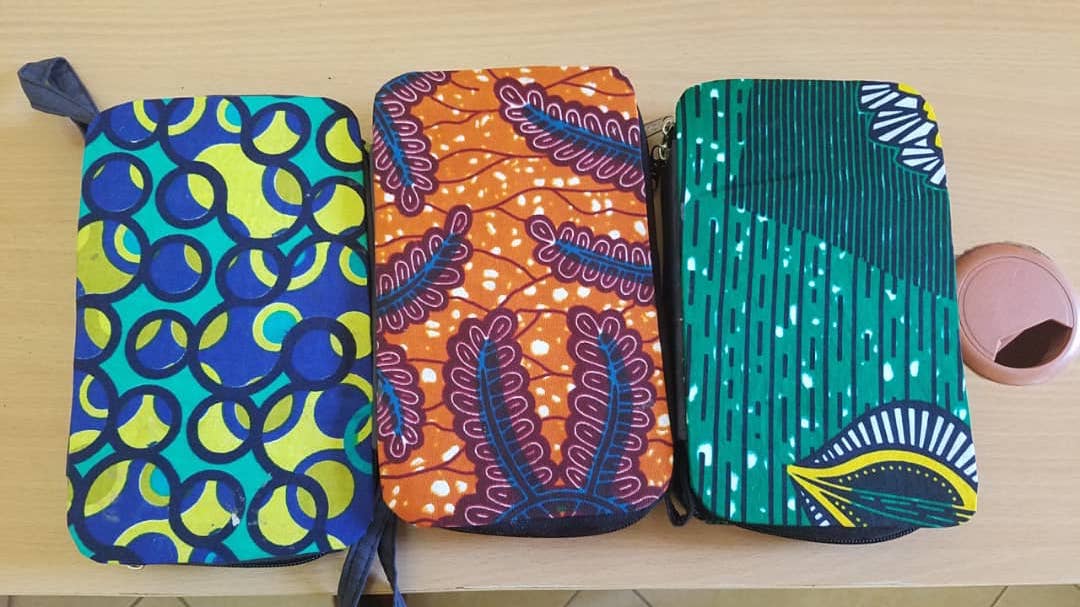

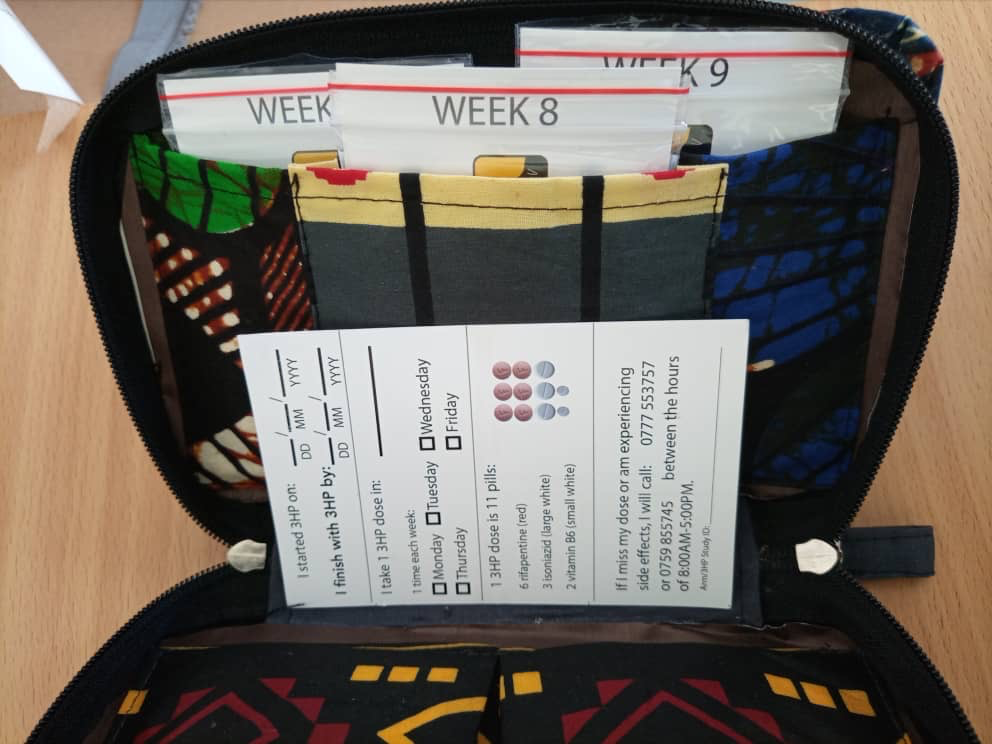


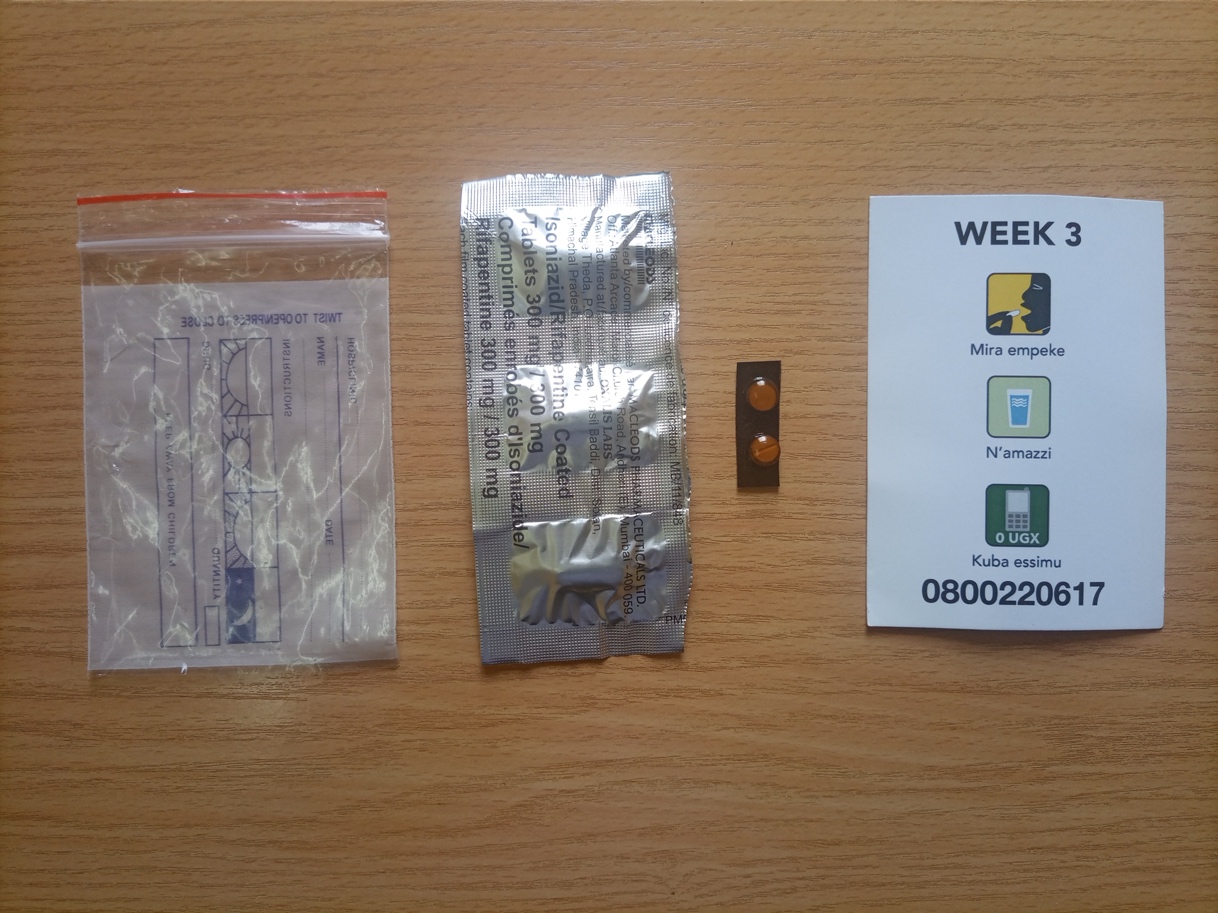

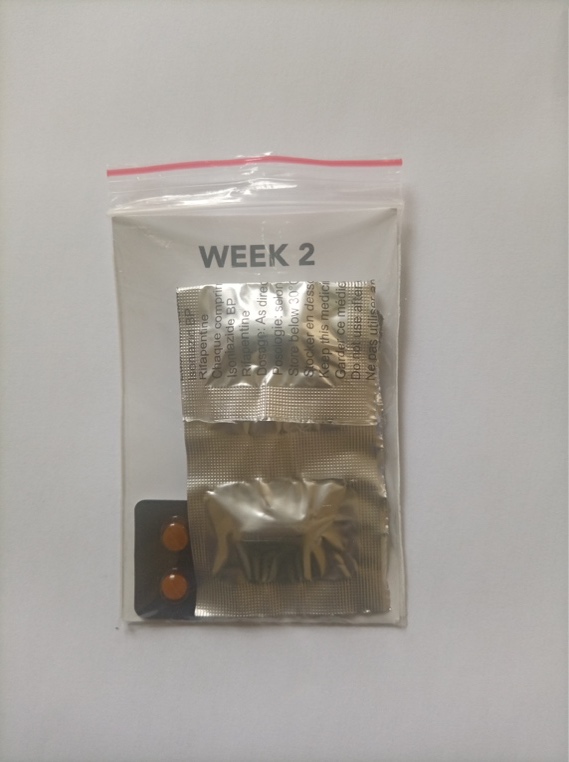


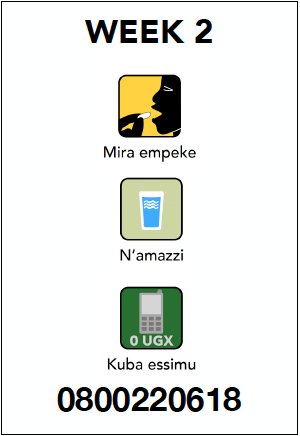

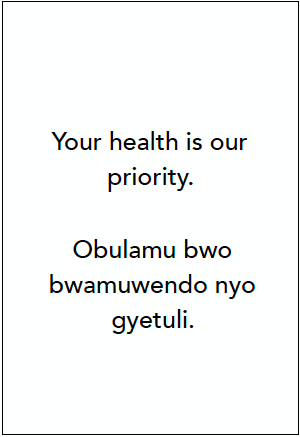

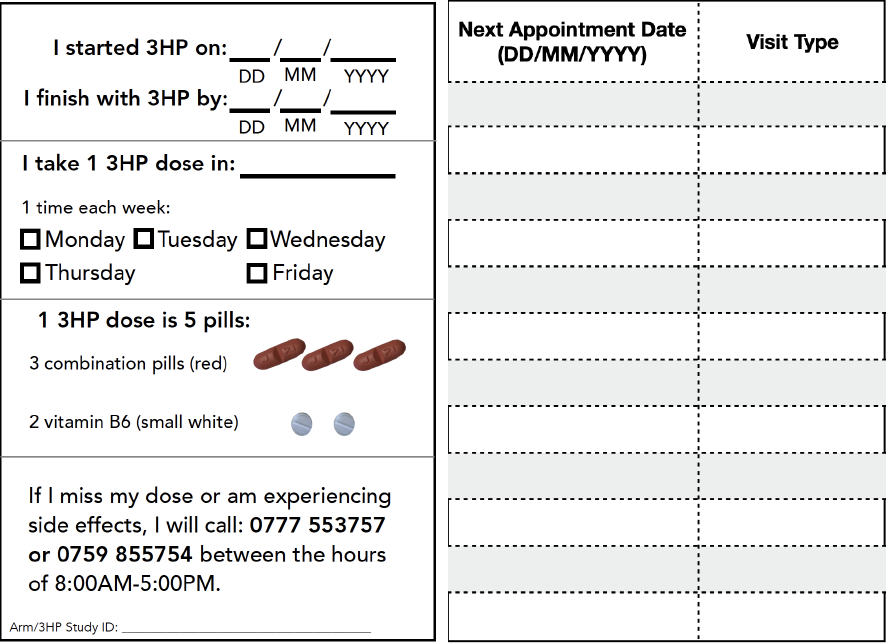

Supplement: S2 Fig — (DOCX) [file pmed.1004356.s004.docx]
