# Supplementary material for: Comparison of 3 optimized delivery strategies for completion of isoniazid-rifapentine (3HP) for tuberculosis prevention among people living with HIV in Uganda: A single-center randomized trial
Source: PLoS Med. 2024 Feb 20;21(2):e1004356. doi: 10.1371/journal.pmed.1004356 (PMC10914279; doi:10.1371/journal.pmed.1004356)
Supplement: S3 Fig — (DOCX) [file pmed.1004356.s005.docx]

**Supplement Figure 3. Subgroup analyses of the primary trial endpoint (acceptance and completion of 3HP).** Panels show pre-specified subgroup analyses of the proportion of participants accepting and competing 3HP, by sex (panel A), age in years (panel B, median 42 years), years on ART (panel C, median 9 years), and prior tuberculosis status (panel D). Point estimates of proportions are represented as solid circles and solid horizontal capped lines depict their corresponding 97.5% confidence intervals. The dotted vertical line at 0.80 represents the pre-specified acceptance and completion threshold against which we assessed each arm.


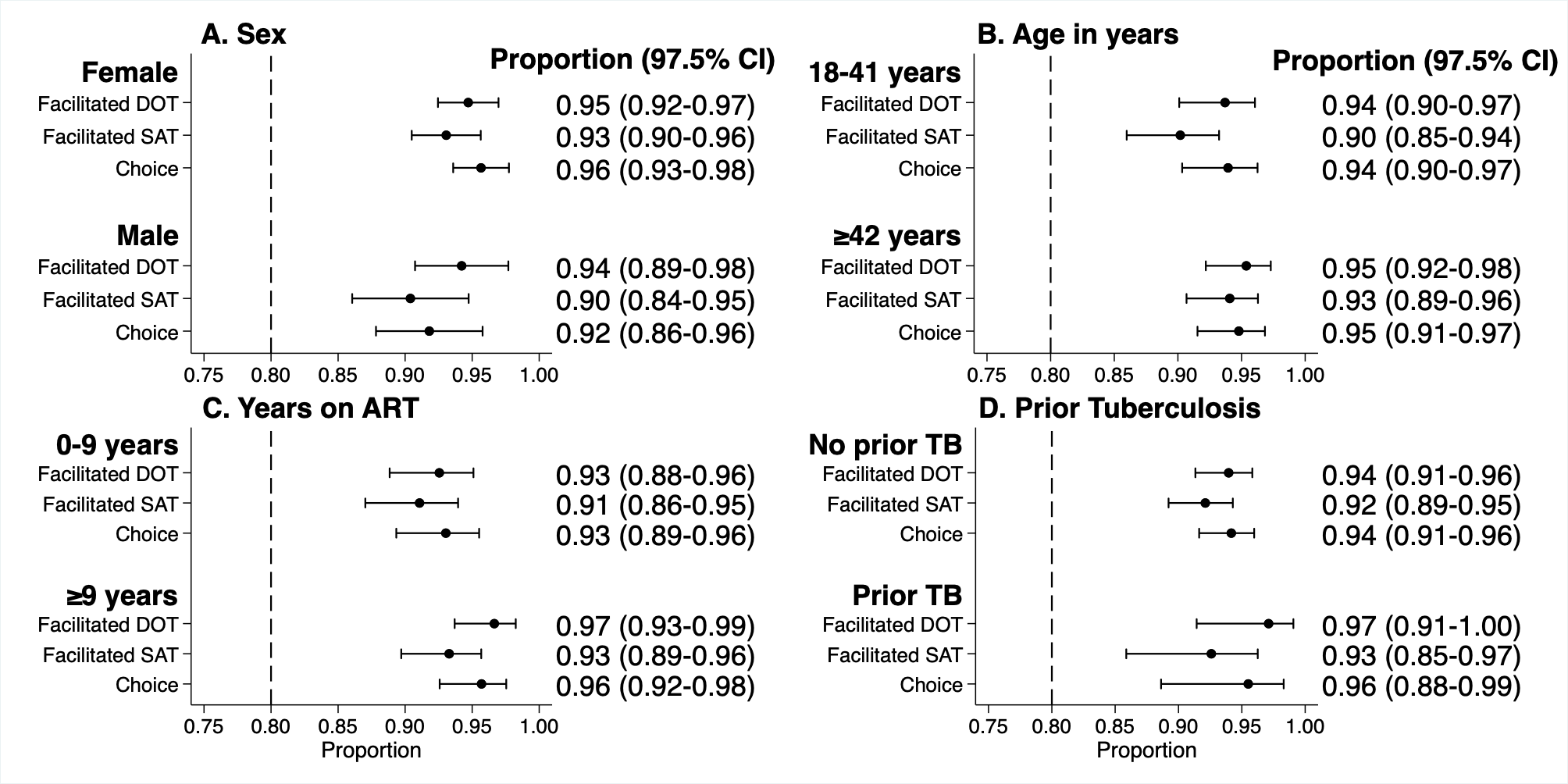


3HP=twelve weeks of once-weekly isoniazid and rifapentine; ART=antiretroviral therapy; CI=confidence interval; DOT=directly observed therapy; SAT=self-administered therapy; TB=tuberculosis
