# Supplementary material for: Comparison of 3 optimized delivery strategies for completion of isoniazid-rifapentine (3HP) for tuberculosis prevention among people living with HIV in Uganda: A single-center randomized trial
Source: PLoS Med. 2024 Feb 20;21(2):e1004356. doi: 10.1371/journal.pmed.1004356 (PMC10914279; doi:10.1371/journal.pmed.1004356)
Supplement: S4 Fig — (DOCX) [file pmed.1004356.s006.docx]

**Supplement Figure 4. Subgroup analyses comparing 3HP acceptance and completion across trial arms.** Comparisons of the trial primary outcome (3HP acceptance and completion) within pre-specified subgroups presented as unadjusted prevalence ratio point estimates (solid circles) and 97.5% confidence interval error bars. Colors indicate the pre-specified subgroups included (red = sex, blue = age, purple = time on ART, green = prior TB status). All 97.5% confidence intervals contained a prevalence ratio of 1.00, indicating no statistically significant difference across any two arms, among any of the pre-specified subgroups evaluated.


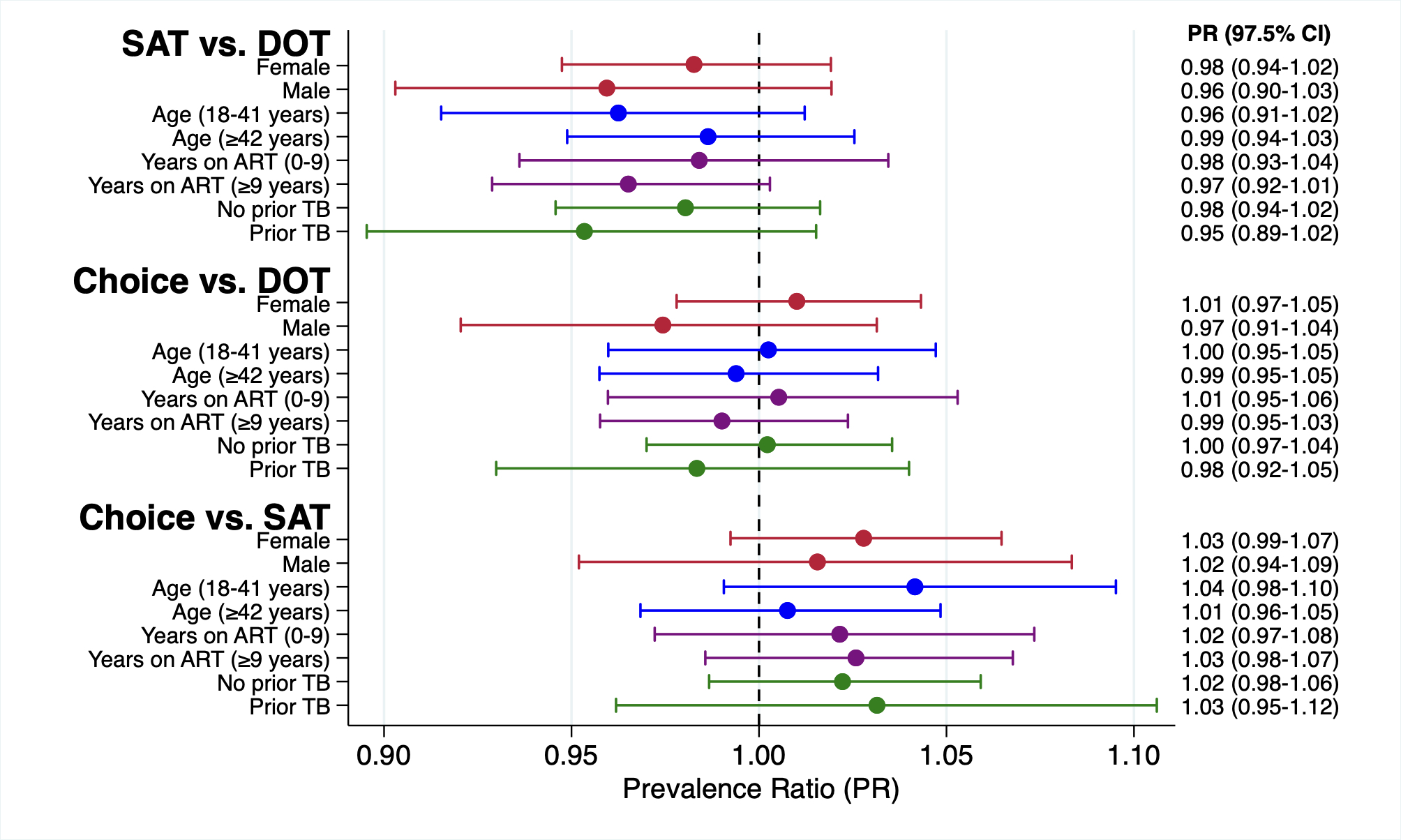


3HP=twelve weeks of once-weekly isoniazid and rifapentine; ART=antiretroviral therapy; CI=confidence interval; DOT=directly observed therapy; PR=prevalence ratio; SAT=self-administered therapy; TB=tuberculosis
