# Supplementary material for: Comparison of 3 optimized delivery strategies for completion of isoniazid-rifapentine (3HP) for tuberculosis prevention among people living with HIV in Uganda: A single-center randomized trial
Source: PLoS Med. 2024 Feb 20;21(2):e1004356. doi: 10.1371/journal.pmed.1004356 (PMC10914279; doi:10.1371/journal.pmed.1004356)
Supplement: S1 Table — (DOCX) [file pmed.1004356.s007.docx]

**Supplement Table 1. Comparison of choice of delivery strategies across key participant characteristics including sex, age, and educational level (N=552)^a,b^.**

|  | CHOICE – DOT  (n=370) | CHOICE – SAT  (n=182) | p-value |
| --- | --- | --- | --- |
| Sex |  |  | 0.03 |
| Female | 236 (63.8) | 133 (73.1) |  |
| Male | 134 (36.2) | 49 (26.9) |  |
| Age | 41.8 (9.4) | 43.2 (9.5) | 0.09 |
| Education |  |  | 0.25 |
| None | 37 (10.0) | 12 (6.6) |  |
| Primary | 181 (48.9) | 85 (46.7) |  |
| Secondary | 126 (34.1) | 64 (35.2) |  |
| Tertiary/Vocational | 17 (4.6) | 16 (8.8) |  |
| University/Graduate School | 9 (2.4) | 5 (2.8) |  |

DOT=directly observed therapy; SAT=self-administered therapy

1. Data are n (%) or mean and standard deviation.
2. A research nurse used a counselling flipbook to provide those who had been randomized to the patient choice arm a brief overview of the facilitated DOT and facilitated SAT delivery strategies. Participants were then asked to state their preferred option for either delivery strategy regarding key concepts related to 3HP delivery, a process that was guided by the shared decision-making tool. The research nurse would engage the participant in a discussion regarding his or her stated preferences and after addressing any questions, ask the participant to select facilitated DOT or facilitated SAT.
