# Supplementary material for: Comparison of 3 optimized delivery strategies for completion of isoniazid-rifapentine (3HP) for tuberculosis prevention among people living with HIV in Uganda: A single-center randomized trial
Source: PLoS Med. 2024 Feb 20;21(2):e1004356. doi: 10.1371/journal.pmed.1004356 (PMC10914279; doi:10.1371/journal.pmed.1004356)
Supplement: S2 Table — (DOCX) [file pmed.1004356.s008.docx]

**Supplement Table 2. Reasons for stopping 3HP treatment.** Reasons for not completing 3HP treatment collected among a sample (n=73) of the 81 participants who did not complete at least 11 of 12 doses of 3HP within 16 weeks of treatment initiation.

|  | N (%) |
| --- | --- |
| The medications were making me feel bad/self-discontinued due to experienced side effects or drug-drug interactions^a^ | 28/73 (38.4%) |
| Transport-related barriers reaching the clinic | 12/73 (16.4%) |
| Inconvenience/stigma/lost interest | 9/73 (12.3%) |
| Relocated or travelled and no longer able to reach the clinic | 8/73 (11.0%) |
| Work-related barriers | 7/73 (9.6%) |
| Misunderstood correct number of doses to take | 6/73 (8.2%) |
| Got into an accident and was unable to continue treatment | 3/73 (4.1%) |

3HP=twelve weeks of once-weekly isoniazid and rifapentine

1. One participant self-discontinued because she wanted to re-start hormonal birth control, which is contraindicated with 3HP.
