# Supplementary material for: Comparison of 3 optimized delivery strategies for completion of isoniazid-rifapentine (3HP) for tuberculosis prevention among people living with HIV in Uganda: A single-center randomized trial
Source: PLoS Med. 2024 Feb 20;21(2):e1004356. doi: 10.1371/journal.pmed.1004356 (PMC10914279; doi:10.1371/journal.pmed.1004356)
Supplement: S3 Table — (DOCX) [file pmed.1004356.s009.docx]

**Supplement Table 3.** Adverse events leading to 3HP discontinuation.

|  | **Facilitated DOT**  **(n=3)** | **Facilitated SAT**  **(n=7)** | **Informed Choice**  **(n=4)** |
| --- | --- | --- | --- |
| **Generalized pruritus^a^** | 2 (66.7%) | 1 (14.3%) | 1 (25.0%) |
| **3HP-related hypersensitivity** | 0 (0.0%) | 2 (28.6%) | 0 (0.0%) |
| **Venous thromboembolism^a^** | 0 (0.0%) | 0 (0.0%) | 1 (25.0%) |
| **Flu-like syndrome** |  |  |  |
| Flu-like syndrome only | 0 (0.0%) | 1 (14.3%) | 0 (0.0%) |
| Flu-like syndrome and peripheral neuropathy | 0 (0.0%) | 0 (0.0%) | 1 (25.0%) |
| **Pulmonary embolism^a^** | 0 (0.0%) | 0 (0.0%) | 1 (25.0%) |
| **Acute liver injury** | 0 (0.0%) | 1 (14.3%) | 0 (0.0%) |
| **Gastritis** | 1 (33.3%) | 0 (0.0%) | 0 (0.0%) |
| **Drug-induced hearing impairment** | 0 (0.0%) | 1 (14.3%) | 0 (0.0%) |
| **Eye disorder^a^** | 0 (0.0%) | 1 (14.3%) | 0 (0.0%) |

3HP=twelve weeks of once-weekly isoniazid and rifapentine; DOT=Directly observed therapy; SAT=self-administered therapy

1. Adverse events classified as “Possibly related” (Generalized pruritis [n=1], venous thromboembolism [n=1], pulmonary embolism [n=1]) or “Unlikely related” (Eye disorder [n=1]). All other adverse events were classified as “Probably related”.
