# Supplementary material for: Comparison of 3 optimized delivery strategies for completion of isoniazid-rifapentine (3HP) for tuberculosis prevention among people living with HIV in Uganda: A single-center randomized trial
Source: PLoS Med. 2024 Feb 20;21(2):e1004356. doi: 10.1371/journal.pmed.1004356 (PMC10914279; doi:10.1371/journal.pmed.1004356)
Supplement: S4 Table — (DOCX) [file pmed.1004356.s010.docx]

**Supplement Table 4.** Pairwise comparisons of treatment discontinuation due to an adverse event as unadjusted odds ratios and unadjusted risk differences with corresponding 97.5% confidence intervals (CI).

|  | **Unadjusted Odds Ratio**  **(97.5% CI)** | **p-value** | **Unadjusted Risk Difference**  **(97.5% CI)** | **p-value** |
| --- | --- | --- | --- | --- |
| **Facilitated SAT vs. Facilitated DOT** | 2.34 [0.50, 11.06] | 0.219 | 0.72% [-0.56%, 2.00%] | 0.206 |
| **Choice vs. Facilitated SAT** | 0.57 [0.14, 2.34] | 0.374 | -0.54% [-1.88%, 0.81%] | 0.368 |
| **Choice vs. Facilitated DOT** | 1.34 [0.24, 7.45] | 0.704 | 0.18% [-0.89%, 1.26%] | 0.703 |

CI=confidence interval; DOT=Directly observed therapy; SAT=self-administered therapy
