# Supplementary material for: Comparison of 3 optimized delivery strategies for completion of isoniazid-rifapentine (3HP) for tuberculosis prevention among people living with HIV in Uganda: A single-center randomized trial
Source: PLoS Med. 2024 Feb 20;21(2):e1004356. doi: 10.1371/journal.pmed.1004356 (PMC10914279; doi:10.1371/journal.pmed.1004356)
Supplement: S5 Table — (DOCX) [file pmed.1004356.s011.docx]

**Supplement Table 5.** **Reimbursement metrics.** Participants who presented for in person clinical visits who were reimbursed successfully.

|  | n (%)  Reimbursed successfully | | | |
| --- | --- | --- | --- | --- |
|  | **Facilitated DOT** | **Facilitated SAT** | **CHOICE** | **Total** |
| Week 2 | 537/546 (98.4) | N/A | 368/368 (100.0) | 905/914 (99.0) |
| Week 3 | 538/541 (99.5) | N/A | 358/358 (100.0) | 896/899 (99.7) |
| Week 4 | 528/539 (98.0) | N/A | 356/356 (100.0) | 884/895 (98.8) |
| Week 5 | 532/536 (99.3) | N/A | 355/355 (100.0) | 887/891 (99.6) |
| Week 6 | 527/535 (98.5) | 522/522 (100.0) | 526/529 (99.4) | 1575/1586 (99.3) |
| Week 7 | 524/532 (98.5) | N/A | 356/356 (100.0) | 880/888 (99.1) |
| Week 8 | 528/530 (99.6) | N/A | 352/353 (99.7) | 880/883 (99.7) |
| Week 9 | 524/528 (99.2) | N/A | 349/349 (100.0) | 873/877 (99.5) |
| Week 10 | 516/525 (98.3) | N/A | 350/350 (100.0) | 866/875 (99.0) |
| Week 11 | 516/521 (99.0) | N/A | 350/350 (100.0) | 866/871 (99.4) |
| Week 12 | 517/520 (99.4) | 508/508 (100.0) | 516/516 (100.0) | 1541/1544 (99.8) |

DOT=directly observed therapy; SAT=self-administered therapy
