# Supplementary material for: Comparison of 3 optimized delivery strategies for completion of isoniazid-rifapentine (3HP) for tuberculosis prevention among people living with HIV in Uganda: A single-center randomized trial
Source: PLoS Med. 2024 Feb 20;21(2):e1004356. doi: 10.1371/journal.pmed.1004356 (PMC10914279; doi:10.1371/journal.pmed.1004356)
Supplement: S6 Table — (DOCX) [file pmed.1004356.s012.docx]

**Supplement Table 6. Time spent on 3HP-related activities.** Time spent in minutes at the Mulago HIV/AIDS Clinic for various study-related activities including clinical visits and completion of the shared decision-making tool activity.

|  | Mean (SD) | Median (IQR) |
| --- | --- | --- |
| Time (minutes) spent at the clinic for a 3HP refill or directly observed therapy (DOT) visit among a sample of participants selected for observation in the time-and-motion sub-study (n=328) | 41.5 (41.2) | 23.5 (11-56) |
| Estimated time (minutes) spent at the clinic for a 3HP refill or DOT visit among all participants with completed endline survey data (n=1638) | 16.3 (17.7) | 10 (5-20) |
| Time (minutes) spent on completing the shared decision-making tool among participants randomized to the Choice study arm (n=552)^a.^ | 10.6 (4.1) | 10 (8-12) |

3HP= twelve weeks of once-weekly isoniazid and rifapentine, SD=standard deviation, IQR=interquartile range

1. A research nurse used a counselling flipbook to provide those who had been randomized to the patient choice arm a brief overview of the facilitated DOT and facilitated SAT delivery strategies. Participants were then asked to state their preferred option for either delivery strategy regarding key concepts related to 3HP delivery, a process that was guided by the shared decision-making tool. The research nurse would engage the participant in a discussion regarding his or her stated preferences and after addressing any questions, ask the participant to select facilitated DOT or facilitated SAT.
