# Supplementary material for: Comparison of 3 optimized delivery strategies for completion of isoniazid-rifapentine (3HP) for tuberculosis prevention among people living with HIV in Uganda: A single-center randomized trial
Source: PLoS Med. 2024 Feb 20;21(2):e1004356. doi: 10.1371/journal.pmed.1004356 (PMC10914279; doi:10.1371/journal.pmed.1004356)
Supplement: S7 Table — (DOCX) [file pmed.1004356.s013.docx]

**Supplement Table 7**. **99DOTS implementation metrics.** Metrics on the implementation of the 99DOTS-based interactive voice response check in calls, appointment reminders, support actions taken in response to missed doses and responses by participants taking 3HP by self-administered therapy (SAT) to interactive voice response check-in calls populated onto task lists hosted on the 99DOTS platform.

| **Interactive Voice Response (IVR) check-in calls** | **n (%)** |
| --- | --- |
| Proportion of IVR check in calls initiated by 99DOTS (SAT only)^a^ | 6150/6693 (91.9) |
| Proportion not connected successfully | 1994/6150 (32.4) |
| Proportion unanswered | 1183/4156 (28.5) |
| Proportion answered but with no response | 1571/4156 (37.8) |
| Proportion answered and with response | 1402/4156 (33.7) |
| Proportion feeling well | 1298/1402 (92.6) |
| Proportion not feeling well | 90/1402 (6.4) |
| Proportion unclear response | 14/1402 (1.0) |
| **Appointment reminders** |  |
| Proportion of appointment reminders sent^b,c^ | 7825/9141 (85.6) |
| **Task Lists** |  |
| Number of items ever populated onto the Missed dose task list | 2,561 |
| Number of patients who ever appeared on the task list | 1023/1655 (61.8) |
| Median # times on task list | 2 (1-3) |
| Number of items ever populated onto the Negative check-in task list | 75 |
| Number of patients who ever appeared on the task list | 63/1655 (3.8) |
| Median # times on task list | 1 (1-1) |
| Number of items ever populated onto the Missed check-in task list | 1,617 |
| Number of patients who ever appeared on the task list | 585/1655 (35.3) |
| Median # times on task list | 2 (2-3) |
| **Support actions** |  |
| Number of phone calls to patients | 1,335 |
| Proportion of patients receiving a phone call | 550/1655 (33.2) |
| Median # calls per patient | 4 (2-5) |
| Number of additional clinic visits completed by patients | 58 |
| Proportion of patients with an additional clinic visit | 53/1655 (3.2) |
| Median # visits per patient | 1 (1-1) |
| Number of home visits completed | 13 |
| Proportion of patients visited at home | 12/1655 (0.7) |
| Median # home visits per patient | 1 (1-1) |

3HP=twelve weeks of once-weekly isoniazid and rifapentine

1. The denominator was calculated based on the total number of expected interactive voice response (IVR) check-in phone calls sent to self-administered therapy (SAT) or Choice-SAT participants prior to doses 2-5 and 7-11. The denominator excludes the number of reminders not sent for doses that participants did not take among those who did not complete 3HP treatment.
2. The denominator was calculated based on the total number of times participants were expected to return to the clinic to attend in-person visits. This included up to eleven visits for directly observed therapy (DOT) participants and Choice-DOT participants, and up to two visits for self-administered therapy (SAT) and Choice-SAT participants. The denominator excludes the number of times visits were not expected for participants who did not complete 3HP treatment.
3. Appointment reminder data only available from February 1, 2021 onward.
