# Supplementary material for: Comparison of 3 optimized delivery strategies for completion of isoniazid-rifapentine (3HP) for tuberculosis prevention among people living with HIV in Uganda: A single-center randomized trial
Source: PLoS Med. 2024 Feb 20;21(2):e1004356. doi: 10.1371/journal.pmed.1004356 (PMC10914279; doi:10.1371/journal.pmed.1004356)
Supplement: S8 Table — (DOCX) [file pmed.1004356.s014.docx]

**Supplement Table 8. Confirmation of self-administered doses via 99DOTS**. Number and proportion of weekly 3HP doses confirmed via toll-free phone calls to 99DOTS platform by participants who were randomized to the self-administered therapy (SAT) arm or who were randomized to the Choice arm and chose SAT as their 3HP delivery method (N=680).

|  | Method: Self-reported via 99DOTS | | |
| --- | --- | --- | --- |
|  | **Facilitated SAT** | **CHOICE – SAT** | **Overall** |
| Dose 2 | 457 (89.6) | 147 (86.5) | 604 (88.8) |
| Dose 3 | 451 (88.4) | 145 (85.3) | 596 (87.7) |
| Dose 4 | 450 (88.2) | 139 (81.8) | 589 (86.6) |
| Dose 5 | 442 (86.7) | 143 (84.1) | 585 (86.0) |
| Dose 7 | 423 (82.9) | 138 (81.2) | 561 (82.5) |
| Dose 8 | 437 (85.7) | 143 (84.1) | 580 (85.3) |
| Dose 9 | 436 (85.5) | 142/ (83.5) | 578 (85.0) |
| Dose 10 | 428 (83.9) | 144 (84.7) | 572 (84.1) |
| Dose 11 | 436 (85.5) | 144 (84.7) | 580 (85.3) |
| OVERALL | 3960 (86.3) | 1285 (84.0) | 5245 (85.7) |

3HP=twelve weeks of once-weekly isoniazid and rifapentine
