# Supplementary material for: Comparison of 3 optimized delivery strategies for completion of isoniazid-rifapentine (3HP) for tuberculosis prevention among people living with HIV in Uganda: A single-center randomized trial
Source: PLoS Med. 2024 Feb 20;21(2):e1004356. doi: 10.1371/journal.pmed.1004356 (PMC10914279; doi:10.1371/journal.pmed.1004356)
Supplement: S9 Table — (DOCX) [file pmed.1004356.s015.docx]

**Supplement Table 9. Patient Satisfaction Survey Results.** Median scores for domains within the patient satisfaction survey administered to participants after they had completed or stopped 3HP treatment (N=1638).

|  | Facilitated DOT  n=546 | Facilitated SAT  n=548 | CHOICE  n=544 |
| --- | --- | --- | --- |
|  | **Median (IQR)** | | |
| How easy/difficult was it for you to travel to this clinic^a^ | 1 (1-1) | 1 (1-1) | 1 (1-1) |
| How easy/difficult was it to find where to go within the clinic to take your next 3HP dose or obtain a refill?^a^ | 1 (1-1) | 1 (1-1) | 1 (1-1) |
| How acceptable/unacceptable was the waiting time to receive services related to 3HP treatment?^b^ | 1 (1-1) | 1 (1-1) | 1 (1-1) |
| How clean/dirty was the clinic?^c^ | 1 (1-1) | 1 (1-1) | 1 (1-1) |
| How comfortable/uncomfortable was the waiting area?^d^ | 1 (1-1) | 1 (1-1) | 1 (1-1) |
| How carefully/uncarefully did the health staff in this facility listen to you regarding any concerns about tuberculosis preventive therapy?^e^ | 1 (1-1) | 1 (1-1) | 1 (1-1) |
| Throughout the course of your 3HP treatment, how satisfied/dissatisfied were you with the amount of time health staff in this facility spent with you?^f^ | 1 (1-1) | 1 (1-1) | 1 (1-1) |
| Throughout the course of your 3HP treatment, how did the health staff treat you compared to other patients?^g^ | 1 (1-1) | 1 (1-1) | 1 (1-1) |
| Throughout the course of your 3HP treatment, how easy/difficult was it to understand explanations about TB preventive care from health staff in this facility?^a^ | 1 (1-1) | 1 (1-1) | 1 (1-1) |
| Throughout the course of your 3HP treatment, how satisfied/dissatisfied were you with the quality of tuberculosis services you received?^f^ | 1 (1-1) | 1 (1-1) | 1 (1-1) |
| Throughout the course of my 3HP treatment, I have not received the best health care as I expected from this clinic.^h^ | 1 (1-1) | 1 (1-1) | 1 (1-1) |
| Throughout the course of your 3HP treatment, how satisfied/dissatisfied were you with the way you were treated by the staff who provided you with tuberculosis services?^f^ | 1 (1-1) | 1 (1-1) | 1 (1-1) |
| Throughout the course of your 3HP treatment, how closely did the tuberculosis preventive care you received from this clinic meet your expectations?^i^ | 2 (1-3) | 2 (1-3) | 2 (1-3) |

3HP=twelve weeks of once-weekly isoniazid and rifapentine, DOT=directly observed therapy; SAT=self-administered therapy; IQR=interquartile range

1. 1=Very easy, 2=Somewhat easy, 3=Neither easy nor difficult, 4=Somewhat difficult, 5=Very difficult
2. 1=Very acceptable, 2=Somewhat acceptable, 3=Neither acceptable nor unacceptable, 4=Somewhat unacceptable, 5=Very unacceptable
3. 1=Very clean, 2=Somewhat clean, 3=Neither clean nor dirty, 4=Somewhat dirty, 5=Very dirty
4. 1=Very comfortable, 2=Somewhat comfortable, 3=Neither comfortable nor uncomfortable, 4=Somewhat uncomfortable, 5=Very uncomfortable
5. 1=Very carefully, 2=Somewhat carefully, 3=Neither carefully nor uncarefully, 4=Somewhat uncarefully, 5=Very uncarefully
6. 1=Very satisfied, 2=Somewhat satisfied, 3=Neither satisfied nor unsatisfied, 4=Somewhat unsatisfied, 5=Very unsatisfied
7. 1=Very similar, 2=Somewhat similar, 3=Neither similar nor different, 4=Somewhat different, 5=Very different
8. 1=Strongly disagree, 2=Somewhat disagree, 3=Neither agree nor disagree, 4=Somewhat agree, 5=Agree, 6=Strongly agree
9. 1=Greatly exceeded expectations, 2=Exceeded expectations, 3=As expected, 4=Below expectations, 5=Much below expectations
