# Supplementary material for: Comparison of 3 optimized delivery strategies for completion of isoniazid-rifapentine (3HP) for tuberculosis prevention among people living with HIV in Uganda: A single-center randomized trial
Source: PLoS Med. 2024 Feb 20;21(2):e1004356. doi: 10.1371/journal.pmed.1004356 (PMC10914279; doi:10.1371/journal.pmed.1004356)
Supplement: S10 Table — (DOCX) [file pmed.1004356.s016.docx]

**Supplement Table 10.** **Responses to Shared Decision-Making Questionnaire.** Participants (n=552) randomized to the choice arm were invited to respond how much they agreed or disagreed with nine statements about the shared decision-making process for choosing between directly observed therapy (DOT) and self-administered therapy (SAT) delivery strategies. The questionnaire was administered directly following the completion of the decision-making process by a different member of the study staff than the person who completed the shared decision-making tool with the participant^a^. Response options ranged from “Completely disagree” to “Completely agree”: 1=Completely disagree, 2=Strongly disagree, 3=Somewhat disagree, 4=Somewhat agree, 5=Strongly agree, 6=Completely agree.

|  | CHOICE – DOT  n=370 | CHOICE – SAT  n=182 |
| --- | --- | --- |
|  | **Median (IQR)** | |
| My counselor made it clear that a decision needs to be made. | 6 (6-6) | 6 (6-6) |
| My counselor wanted to know exactly how I want to be involved in making the decision. | 6 (6-6) | 6 (6-6) |
| My counselor told me that there are different options for how to receive 3HP treatment. | 6 (6-6) | 6 (6-6) |
| My counselor clearly explained the advantages and disadvantages of the treatment options. | 6 (6-6) | 6 (6-6) |
| My counselor helped me understand all the information. | 6 (6-6) | 6 (6-6) |
| My counselor asked me which treatment option I prefer. | 6 (6-6) | 6 (6-6) |
| My counselor and I thoroughly weighed the different treatment options. | 6 (6-6) | 6 (6-6) |
| My counselor and I selected a treatment option together. | 6 (6-6) | 6 (6-6) |
| My counselor and I reached an agreement on how to proceed. | 6 (6-6) | 6 (6-6) |

IQR=interquartile range

1. A research nurse used a counselling flipbook to provide those who had been randomized to the patient choice arm a brief overview of the facilitated DOT and facilitated SAT delivery strategies. Participants were then asked to state their preferred option for either delivery strategy regarding key concepts related to 3HP delivery, a process that was guided by the shared decision-making tool. The research nurse would engage the participant in a discussion regarding his or her stated preferences and after addressing any questions, ask the participant to select facilitated DOT or facilitated SAT.
