# Supplementary material for: Comparison of 3 optimized delivery strategies for completion of isoniazid-rifapentine (3HP) for tuberculosis prevention among people living with HIV in Uganda: A single-center randomized trial
Source: PLoS Med. 2024 Feb 20;21(2):e1004356. doi: 10.1371/journal.pmed.1004356 (PMC10914279; doi:10.1371/journal.pmed.1004356)
Supplement: Attachment — Submitted filename: Response to Reviewers.docx [file pmed.1004356.s019.docx]

**Comments from the academic editor:**
It is an excellent paper but in order for this to not be just more data compared to before, further sub-analysis would be valuable.

**Comments from the editorial team:**
We are much in agreement with the academic editor and reviewer #2 that the secondary outcomes should be reported here; particularly given the noted study limitations and that the full results are similar to the interim analysis. We offer major revisions contingent on presentation of secondary outcomes.

- **Response:** Thank you very much for your review of our manuscript. We would like to highlight that the interim analysis presented aggregated results across arms whereas the trial-arm specific results reported here provide important new information. In particular, the finding that 3HP can be delivered successfully by self-administered therapy (SAT) (with appropriate facilitation) in the context of routine HIV care is new and has important implications for scale-up. However, based on the recommendations from the academic editor, the editorial team, and the reviewers, we have included in the revised manuscript and in the new Supplemental data (Supplement 4) additional analyses of secondary outcome data that we believe addresses these concerns. We thank the reviewers and the editorial team for the opportunity to improve the manuscript.

**GENERAL**
Please provide 95% CIs and p values for all results where appropriate (including the abstract), check and amend throughout. We suggest reporting statistical information in the following format: ‘x’; (95% CI [‘y’,’ z’] p value) and use commas as opposed to hyphens (as these can be confused with negative values) to separate upper and lower bounds.

- **Response:** Thank you, we have edited the document to be in accordance with the reporting guidelines for statistical information as described above.

For p values, please report as p<0.001 and where higher as 'p=0.002'. Please add the statistical method used to your method section. We also invite you to report p values to consistently to the third decimal digit - thousandths.

- **Response:** Thank you, we have updated the p-values to be reported to the third digit throughout the manuscript as well as in Supplement Table 1. Statistical methods can be found in paragraph 2 of the methods section, and the detailed statistical analysis plan is provided in Supplement 3.

For in-text reference, citations are placed within square parentheses and should precede punctuation. Please amend throughout. (e.g line 107-108 should be: ‘Tuberculosis (TB) is a curable and preventable disease but remains a leading cause of death worldwide, especially among people living with HIV (PLHIV) [1].) Please amend throughout.

- **Response:** Thank you, we have corrected the in-text reference style throughout the document.

**STUDY DESIGN:**

Please complete the CONSORT checklist and ensure that all components of CONSORT are present in the manuscript. When completing the checklist, please use section and paragraph numbers, rather than page numbers.

- **Response:** We have included a CONSORT checklist with the submission and included the section and paragraph where the specific information can be found in the manuscript.

**TITLE:**

Please add locality (e.g. Uganda, or Kampala, Uganda) in the title. Please also specify in the title that this study is a single centre study.

- **Response:** We have edited the title of the paper to now be: “Comparison of three optimized delivery strategies for completion of Isoniazid-Rifapentine (3HP) for tuberculosis prevention among people living with HIV in Uganda: a single-center randomized trial

**ABSTRACT**

1. Please report your abstract according to CONSORT for abstracts,

<https://urldefense.com/v3/__https://www.equator-network.org/reporting-guidelines/consort-abstracts/__;!!LQC6Cpwp!t06dNFwMp4eIrf31_2FSeC0WiZzXzlZKdyiAjfA9-l2tMbkWMm_fndXixNixrxpEYNJED9GckSgmlPOHZAHxuYguCQ$> and following the PLOS Medicine abstract structure (Background, Methods and Findings, Conclusions). Please remove all other subheaders.

- **Response:** Thank you, we have edited the abstract to now include the sub-headers of Background, Methods and Findings, and Conclusions.

2. Abstract Methods and Findings: Please include in the study design that the study is single centre.
   - **Response:** We have edited the first sentence of the Methods and Findings section to be the following: *“In a pragmatic, single-center, three-arm, parallel-group randomized trial, PLHIV receiving care at a large urban HIV clinic in Kampala, Uganda, were randomly assigned (1:1:1) to receive 3HP by facilitated directly observed therapy (DOT), facilitated self-administered therapy (SAT), or informed choice between facilitated DOT and facilitated SAT using a shared decision-making aid.”*
3. Please quantify the main results (with 95% CIs and p values).
   - **Response:** We have added the p-values for the main result of 3HP acceptance and completion exceeding 0.80 in the three study arms. We have maintained the 97.5% Bonferroni corrected confidence intervals as originally reported, as we specified in our trial statistical analysis plan that: *“Whether 80% acceptance and completion is achieved will be based on the lower bound of the Bonferroni-corrected 97.5% CI exceeding 0.80 in any of the three arms. We will reject our null hypothesis if the lower bound of the 97.5% CI does not exceed 80%.”*
4. In the last sentence of the Abstract Methods and Findings section, please describe the main limitation(s) of the study's methodology.

- **Response:** Thank you for this suggestion; we have added in the following sentence to the end of the “Methods and Findings” section: *“Multicenter studies are now needed to confirm the feasibility and generalizability of the facilitated 3HP delivery strategies in other settings.”*

**AUTHORS SUMMARY**

1. At this stage, we ask that you include a short, non-technical Author Summary of your research to make findings accessible to a wide audience that includes both scientists and non-scientists. The Author Summary should immediately follow the Abstract in your revised manuscript. This text is subject to editorial change and should be distinct from the scientific abstract. Please see our author guidelines for more information:

<https://urldefense.com/v3/__https://journals.plos.org/plosmedicine/s/revising-your-manuscript*loc-author-summary__;Iw!!LQC6Cpwp!t06dNFwMp4eIrf31_2FSeC0WiZzXzlZKdyiAjfA9-l2tMbkWMm_fndXixNixrxpEYNJED9GckSgmlPOHZAETYDtvkg$>

2. Ideally each sub-heading should contain 2-3 single sentence, concise bullet points containing the most salient points from your study.
3. In the final bullet point of ‘What Do These Findings Mean?’ Please include the main limitations of the study in non-technical language.
   - **Response:** We have added in an “Author Summary” according to the instructions above immediately following the Abstract section of the manuscript.

**ACKNOWLEDGMENTS/ DECLARATIONS**

1. Please remove all statements apart from acknowledgements, author contributions and abbreviations from the end of the main manuscript and include these only in the relevant parts of the manuscript submission form. Funding, competing interest, and data availability will be compiled as metadata.

- **Response:** We have removed the indicated sections that will be compiled as metadata and added in the author contributions per the CRediT Taxonomy guidelines.

**SUPPORTING INFORMATION** has not been checked by the editor.

**Reviewer #1:** "Comparison of three optimized delivery strategies for completion of Isoniazid-Rifapentine (3HP) for tuberculosis prevention among people living with HIV: a randomized trial" reports the outcome of a pragmatic three-arm parallel-group randomized trial with about 550 participants in each arm, on acceptance (and adherence) to short-course tuberculosis (TB) prevention treatment, through either of directly observed therapy (DOT), facilitated self-administered therapy (SAT) or an informed choice between DOT and SAT. The main finding was that all three regimens had high acceptance and completion rates (>0.92). While generally clearly written and extensively documented with implications for TB prevention administration, some issues might be considered:

1. In Line 136, a number of exclusion criteria are stated. In particular, the exclusion for people <40kg might be explained further. What happened to would-be participants weighing less than 40kg?
   - **Response:** Thank you for this comment. Similar to the iAdhere trial

(<https://www.acpjournals.org/doi/epdf/10.7326/M17-1150>), we excluded people who weighed <40 kilograms as 3HP dosages were fixed and not weight adjusted. Ultimately, this criterion likely had limited impact on trial results as only one participant was excluded for being <40 kg (please see Figure 1). Nonetheless, we have edited the information included in Supplement 1 where we describe trial inclusion/exclusion criteria to further explain this rationale.

2. In Line 213, the fidelity rate is defined as the completion of at least 11 of 12 doses of 3HP within 16 weeks of treatment initiation. The definition might be briefly justified.
   - **Response:** This was the definition of treatment completion used in the original PREVENT TB trial that established the efficacy of 3HP, and the definition used in both previous 3HP implementation trials. We now reference these trials when stating the definition used.
3. In Line 223, a minimum clinically important difference of 10% is stated. It might be briefly clarified if this was established by custom, empirical justification or prior literature etc.
   - **Response:** We chose a minimum clinically important difference of 10% based on the only prior implementation study of 3HP delivery strategies that had been completed at the time of trial design. Specifically, the CDC TBTC iAdhere study used a 15% non-inferiority margin between DOT and SAT based on cost-effectiveness modeling in the US. Here, we chose a 10% difference to be more conservative because similar modeling studies have not been done in low-income settings. We have edited the line in the manuscript to be as follows: *“We estimated sample size based on a minimum clinically important difference of 10% in 3HP acceptance and completion between DOT and informed choice arms, which is consistent with prior implementation trials of 3HP delivery strategies (7).”*
4. In Line 231, the Supplement number might be stated.
   - **Response:** Thank you, we have edited to reference the statistical analysis plan as Supplement 3.
5. In Line 271, it is stated that "...73 participants who initiated 3HP missed six or more doses and were classified as not reaching the primary outcome (unable to complete 11 doses within 16 weeks)". The discrepancy between the actual applied condition (missed six or more doses) and the original definition (complete 11 [of 12 doses, from Line 213]) might be explained.
   - **Response:** Thank you for allowing us to clarify. Participants who missed 6 or more weeks of 3HP medication by the time they reached the date 16 weeks since treatment initiation (the allotted time for treatment completion) could at a maximum only have taken 10 total 3HP doses, and thus would not have reached the minimum number of 11 doses within 16 weeks required to attain the primary outcome. However, we recognize the text was confusing and have tried to clarify as follows: *“Among the 1,655 PLHIV included in the primary analysis, 81 (4.9%) participants who initiated 3HP completed fewer than 11 doses within the allotted 16-week treatment period and were thus classified as not reaching the primary outcome”.*
6. Participant-reported reasons for missing multiple doses might be briefly discussed in the Discussion section, if possible.
   - **Response:** Thank you for this suggestion. We have added a Supplemental Table (Supplement 4) with the survey responses from a sample (n=73) of the 81 participants who did not complete 3HP treatment with their reasons for not completing treatment, added a sentence describing these data in the Results section, and edited the following sentence in the Discussion section: *“Survey data reflects that the barriers related to cost and transportation remained a challenge for some participants who were unsuccessful in completing 3HP. However, the overall high level of 3HP acceptance and completion highlights the importance of identifying barriers through formative work and addressing those barriers during implementation.”*
7. In Line 347, it is stated that the adherence rate for WHIP3TB might be suggested to be lower than estimated by completion rate, once pill count was considered. Then, it is stated that "...we demonstrate high levels of 3HP completion by SAT facilitated by the 99DOTS adherence technology". It might be clarified as to whether the 99DOTS technology addresses the issue of adherence rate not complementing the completion rate, i.e. how doe 99DOTS solve the problem of pills/doses reported as being taken, but not actually taken?
   - **Response:** The reviewer is correct that 99DOTS does not solve the problem of pills/doses reported as being taken but not actually taken. We have revised this section of the Discussion as follows: *“In our trial, completion rates were comparable to those achieved in the WHIP3TB trial conducted in South Africa (90.5%), Ethiopia (95.4%), and Mozambique (82.2%), where trained research staff administered 3HP (8). In contrast, our completion rate was much higher than the 50% achieved with SAT in the South African iAdhere trial site (7). However, it is possible in all three trials that actual adherence was lower with SAT as pill ingestion was not confirmed. A subgroup analysis of the WHIP3TB trial data, focusing on those who received 3HP facilitated by electronic medication monitoring, found lower treatment completion (83.5%) than the overall completion rate assessed by pill count alone (8). In our trial, adherence was assessed by both self-report via the 99DOTS platform and pill counts at weeks 6 and 12.”*
8. The calculation/data sources (e.g. from surveys?) for the costings reported in Figure 4 might be briefly described.
   - **Response:** We have added in additional information about the costing methods in both the “Data collection” and “Statistical Analysis” sections of the Methods:
     i. *“Both study and clinic staff logged weekly time requirements for their duties, and clinical activities were observed using time-and-motion surveys. Time data were transferred to human resource cost based on the national average level of each type of staff’s hourly salary. Study staff completed monthly budgetary reviews of bills and receipts for resource-use data including overhead costs, study drugs, drug import fees, office supplies, internet charges, and lab consumables. Cost of transport reimbursement was calculated based on the total number of patients and their number of visits in each arm.”*
     ii. *“We estimated the total per patient cost of each delivery strategy from the health system perspective by compiling cost data from time and motion surveys, weekly time logs and monthly budgetary reviews. Costs were estimated in 2021 Ugandan Shillings and converted to US dollars using the average exchange rate in 2021 (1 USD=3,587.05 USh) (16).”*

**Reviewer #2:** The authors conducted a randomized controlled implementation science trial to determine the optimal delivery strategies for TB preventive therapy (3HP) in Uganda. The team had two distinct intervention arms, and a third trial arm that allowed people to make an informed choice.

1. Overall, the acceptance and completion rates were high, and there were no differences among the three groups. The authors have already presented and published an analysis of acceptance and completion rates for this clinical trial cohort (PLoS Med. 2021/12/17 ed. 2021; Dec;18(12):e1003875). The authors should comment on whether and how the presentation/publication of the interim results could have influenced the remainder of the trial.
   - **Response:** We do not believe presentation/publication of the interim results significantly influenced the remainder of the trial. To minimize impact on the ongoing trial and maintain investigator and study team blinding, the interim analysis focused on the proportion of trial participants completing 3HP treatment **aggregated across study arms**. No trial arm-specific data was analyzed or reported in the interim analysis, and trial investigators and staff remained blinded to trial arm-specific results. Of note, the final trial results aggregated across arms were nearly identical to the aggregate results reported in the interim analysis (92.9% vs. 93.7% treatment completion across arms). Moreover, we now report there were no differences between trial arms, which further supports that publication of aggregated trial data as interim results was unlikely to have influenced the remainder of the trial. To address the reviewer’s important comment about this, we have added the following sentence to the Discussion: *“Although we conducted and reported an interim analysis of the primary outcome aggregated across arms (20), the minimal difference in the aggregate proportion accepting and completing 3HP in the interim vs. final analysis (0.93 vs. 0.94, respectively) and the lack of difference between trial arms suggest that the interim analysis did not have a major impact on final trial results.”*
2. In addition, the 3HP adoption, implementation, and outcomes measures of TB incidence at one-year are not being reported in this paper.  Since the interim analyses showed high levels of acceptability and completion, these secondary outcomes would be helpful to be reported in this paper to help inform programmatic decisions for 3HP delivery programs. Currently, the additional analyses, stratified by study arm, provide limited additional value.
   - **Response:** Thank you for this suggestion. We believe the trial-arm specific results provide important new information. In particular, the finding that 3HP can be delivered successfully by SAT (with appropriate facilitation) in the context of routine HIV care is new and has important implications for scale-up. However, based on the reviewer’s suggestion, we have now included the quantitative implementation outcomes outlined in the trial protocol, including fidelity and acceptability, which can be found in Supplement 4 and in final paragraph of the Results section of the manuscript. We believe that qualitative and cost-effectiveness outcomes (which require specialized analytic methods and thus more methodological detail) are best presented separately. TB incidence outcome data, including at one year and two years post-3HP completion, are still being actively collected.
3. A major limitation of the study is being a single-site clinical study at one urban HIV clinic in Kampala.  Given this is an IS study, using results from a single trial site to extrapolate programmatic approaches to the rest of Uganda and/or beyond can be problematic.  The rather high treatment completion rates observed in this study also raises concerns that the population (and outcomes) may not be representative of other populations receiving 3HP. The authors could expand on how these results may be relevant (or not) to other settings. The study did not include a control group that was not receiving adherence support. Therefore, differences above the baseline acceptance and completions rates were not assessed. Thus, the study does not address whether adherence support is still needed with the 3HP regimen.  This should be addressed in the Discussion section.
   - **Response:** We appreciate this concern and do not want to overstate the generalizability of our findings to other settings. That being said, the trial site is the largest HIV clinic in Uganda and is representative of urban PEPFAR-funded HIV/AIDS programs in Uganda and sub-Saharan Africa. Completion of TPT with daily isoniazid was known to be low at the trial site. A survey conducted by our team in July 2019 showed that completion of isoniazid preventive therapy (IPT) was 56%. Although it is possible short-course TPT with 3HP alone could dramatically improve treatment completion, this was not seen in the only iAdhere trial site in sub-Saharan Africa. These factors suggest – though admittedly do not prove – that the high completion seen in this pragmatic trial were due to the intervention strategies themselves. Nonetheless, we agree that the facilitated delivery strategies assessed here should be evaluated in other settings. We have highlighted this point more explicitly in the Discussion section : *“This was a single center trial that excluded people living further than 25 kilometers from the clinic and those who did not own/have access to a phone. As with any implementation trial, the tested strategies are likely not to be feasible to implement for all individuals in all settings. For example, individuals without cell phones cannot participate in cell phone-based reminder systems; similarly, people who live very far from a clinic are unlikely to be able to participate in DOT. As such, our findings of high acceptance and completion should be interpreted as reflective of what might be achievable among people who could participate in these interventions, not among all PLHIV eligible for TPT in any given clinic. Similarly, some of the delivery strategy components we evaluated (such as transport reimbursements and IVR phone calls) may not be considered feasible in other clinics. However, broader consideration of such costs (which are often lower than the costs of drugs) in health budgets is needed for effective implementation of novel interventions. Lastly, while the barriers to 3HP completion targeted here are likely to be relevant in many settings, further contextual adaptation may be required for facilitated 3HP implementation.”*
4. Another limitation was the reliance on pill counting among the SAT group. Since obtaining a pill from a pack may not equate to pill ingestion, this outcome was not as rigorous as other possible measurements. However, given the lack of outcome difference, this issue was unlikely to have been problematic in this cohort.

**Response:** We agree with the reviewer. We have edited the Discussion section to further emphasize the limitations of self-reported data in order to address this comment, as well as another reviewer’s comment above: *“In our trial, completion rates were comparable to those achieved in the WHIP3TB trial conducted in South Africa (90.5%), Ethiopia (95.4%), and Mozambique (82.2%), where trained research staff administered 3HP (8). In contrast, our completion rate was much higher than the 50% achieved with SAT in the South African iAdhere trial site (7). However, it is possible in all three trials that actual adherence was lower with SAT as pill ingestion was not confirmed. A subgroup analysis of the WHIP3TB trial data, focusing on those who received 3HP facilitated by electronic medication monitoring, found lower treatment completion (83.5%) than the overall completion rate assessed by pill count alone (8). In our trial, adherence was assessed by both self-report via the 99DOTS platform and pill counts at weeks 6 and 12.”*

5. The study appeared to use a composite outcome of reach and fidelity.  The definition for the acceptance component was defined in Figure 2, but should be moved to the Methods section.  Furthermore, reporting an outcome of acceptance is not particularly helpful for an RCT with informed consent (as evidenced in Figure 2).  This outcome could be removed from Figure 2, Fig 3, and elsewhere.
   - **Response:** Thank you for the suggestion; we have edited the “Outcomes” section of the Methods to further clarify the definition of treatment acceptance. However, given that 3HP acceptance was one of our pre-specified secondary outcomes and a key outcome along the RE-AIM domains, we would like to keep the figures as is with 3HP acceptance included. Specifically, the informed consent document told participants that it was their choice whether they wished to actually take 3HP treatment via the delivery strategy to which they were randomized (DOT, SAT, or Choice), even after agreeing to participate in the trial. Thus, for example, a potential participant who would have been willing to take 3HP by SAT but not DOT would have been counseled to enroll in the trial and make a decision about starting 3HP based on their allocation. The rationale for this outcome was to assess whether willingness to take any dose of 3HP via a given delivery strategy differed across trial arms (e.g., if any one of the three delivery strategies was unacceptable compared to the others). Once randomized, participants were included in the denominator for each arm whether or not they accepted to initiate 3HP. Overall, we believe that the high proportions of trial participants who were randomized to a given strategy and took at least one dose across all arms is useful information for demonstrating the high acceptability of 3HP via all facilitated delivery strategies evaluated.
6. Similarly, The 'informed choice' column can be removed from Figure 4, since this is a weighted average of participants who joined the 2 interventions.  While this may be a representative cost for this study arm, it does not appear to represent raw data collected within the choice intervention
   - **Response:** Thank you for this comment. We collected raw data from participants in all three arms. The data were collected by staff who were masked to participants’ trial arm allocation (i.e., they could identify whether a participant was receiving TPT by DOT or SAT, but they did not know whether someone receiving DOT was in the DOT or Choice arms). Moreover, the “informed choice” column does include some additional costs (e.g., the cost of developing the flipbook itself and the time spent going through the choice procedure). The reviewer is correct, however, that these costs were minimal relative to other costs. Given the explanation above, we prefer to leave the 'informed choice' column in Figure 4. But if the reviewer or editors feel strongly, we are happy to remove it.
7. Overall, there was a high rate of exclusion for people not residing in the local area. This likely had an impact on the high completion rates, and also limits generalizability of study findings.  The impact of this exclusion criteria and the impact of interpretation should be discussed.

- **Response:** We excluded people not residing in the local area to avoid creating a bias in favor of SAT (people who live outside the local area are less likely to be willing to return to clinic weekly for DOT). We agree that this could have contributed to the high completion rate in the DOT arm and to the surprisingly high proportion of participants who chose DOT in the Choice arm. But we think this is less likely to impact the high completion rates in the SAT arm and further lends credence to SAT as a more viable option for a broader population of clinic clients. We have now noted this in the Discussion: “*The cost difference between DOT and SAT would likely be even higher if people living >25 kilometers of the study clinic were included in the trial, and incorporating participant-level costs (e.g., lost wages, childcare requirements for clinic visits) is likely to only further support facilitated SAT as a cheaper option than facilitated DOT.”*

**Reviewer #3:** I commend the authors on conducting a well-designed, thoughtful and clinically relevant prospective 3-arm type 3 hybrid effectiveness-implementation study into three delivery strategies (DOT, self-administered therapy or informed choice) for 3HP treatment of latent TB integrated into the workflow of a single large outpatient clinic serving persons with HIV in Kampala, Uganda. I agree with the authors that making shorter effective TB preventative therapy (latent TB) regimens such as 3HP more widely available among persons with HIV in high burden settings  is a key component of the END TB strategy, and as noted by the authors in the introduction and discussion sections previous studies including iAdhere and WHIP3TB have found variable rates of treatment completion with regard to self-administered 3HP in other clinical settings in Africa. Among the 1655 participants in the primary analysis, those in all 3 arms achieved very high rates of acceptance and completion of 3HP treatment.

1. The inclusion criteria and exclusion criteria are clearly laid out. Persons with HIV who started ART within the preceding 3 months were excluded and the authors should specify why this cutoff was used -- I am slightly unclear as to whether the concern was for drug interactions between rifapentine and ART or for IRIS/unmasking of opportunistic infections as potential confounders of study outcomes.
   - **Response:** Thank you for this comment. This was the recommended approach according to the February 2020 Consolidated Guidelines for the Prevention and Treatment of HIV and AIDS by the Ministry of Health in Uganda; *“For newly identified patients, start on dolutegravir (DTG) based antiretroviral therapy with active symptomatic monitoring for adverse events. Initiate TPT after 3 months to allow time for potential unmasking of TB and to monitor any toxicities that may arise from DTG, prior to initiation of TPT.”*

Please see page 99 in the guideline document linked below:

<https://elearning.idi.co.ug/wp-content/uploads/2022/05/Consolidated-Guidelines-for-the-Prevention-and-Treatment-of-HIV-and-AIDS-in-Uganda-2020.pdf>

2. The exclusion of otherwise eligible participants who either did not have a cellphone to receive SMS reminders/automated calls and the exclusion of participants living >25km from the clinic do limit the generalizability of the findings and procedures used with regard to other settings in sub-Saharan Africa, where patients may need to travel long distances to receive care and where availability of cellphone coverage can vary, especially in rural settings. While the authors hint at this in the comments about generalizability to rural settings in the discussion section, I think these are important considerations that would strengthen the discussion. Additionally, I note from the protocol that only those participants who could consent in Luganda or English were eligible, and that another limitation in terms of implementing the phone-based reminder strategies used in this research study more broadly in other resource limited settings may be the need for materials in multiple languages and strategies to mitigate barriers based on literacy. This is where I feel that the single center approach used is something of a limitation, as implied by the authors in the penultimate paragraph of the discussion. The authors do acknowledge that reimbursement of travel and some of the other strategies to mitigate barriers to treatment completion may not be feasible in other settings and that the the barriers may differ in different settings.

**Response:** Thank you, in order to address this comment as well as others from Reviewer #2, we have edited the limitation section of the Discussion to read as follows: *“This was a single center trial that excluded people living further than 25 kilometers from the clinic and those who did not own/have access to a phone. As with any implementation trial, the tested strategies are likely not to be feasible to implement for all individuals in all settings. For example, individuals without cell phones cannot participate in cell phone-based reminder systems; similarly, people who live very far from a clinic are unlikely to be able to participate in DOT. As such, our findings of high acceptance and completion should be interpreted as reflective of what might be achievable among people who could participate in these interventions, not among all PLHIV eligible for TPT in any given clinic. Similarly, some of the delivery strategy components we evaluated (such as transport reimbursements and IVR phone calls) may not be considered feasible in other clinics. However, broader consideration of such costs (which are often lower than the costs of drugs) in health budgets is needed for effective implementation of novel interventions. Lastly, while the barriers to 3HP completion targeted here are likely to be relevant in many settings, further contextual adaptation may be required for facilitated TPT implementation.”*

3. Overall, while this study failed to show that informed choice performed significantly better than DOT or self-administered therapy for 3HP in this setting, the authors clearly demonstrate in their results that self-administered therapy with 3HP is associated with high rates of acceptance and completion in a routine HIV clinic setting in sub-Saharan Africa. I look forward to the promised additional analyses of secondary endpoints, such as TB incidence at 1 year and cost-effectiveness analysis as mentioned in the 'Outcomes' paragraph of the methods section.
   - **Response:** We thank the reviewer for their positive review. We look forward to reporting on the additional outcomes in forthcoming manuscripts.

**Reviewer #4:** I appreciate the opportunity to review this manuscript. The assessment of delivery strategies to improve the acceptance and completion of preventive therapy in people living with HIV is an incredibly important topic being studied in a high-risk population. I praise the use of human centered design methods and person-centered care principles for the optimization of delivery of 3HP. The high acceptance and completion of 3HP is incredibly promising, especially because of the authors' work done locally to integrate these delivery strategies into routine care and the potential for scale-up. I applaud the authors for an incredibly well-written manuscript that is clear to follow and thorough in its description. All tables and figures are clear and concise. While I fully support this manuscript for publication, I have a few minor suggestions for the final version:

1. Did the authors assess completion results separately for individuals who received loose pills early on in the trial vs fixed-dose combination pills later in the trial? Do they anticipate any difference in acceptance or completion based on this?
   - **Response:** Thank you for this suggestion. We switched from loose pills to fixed-dose combination pills once they were available because pill burden was frequently raised as a concern in our formative work. Although numbers are small, we conducted sensitivity analyses assessing: 1) the overall proportion of patients accepting and completing 3HP across arms comparing those enrolled before and after fixed-dose combination (FDC) pills were offered, and 2) the proportion accepting and completing 3HP by study arm and disaggregated by time period during which loose pills vs FDCs were offered. We found no statistically significant difference in the overall completion proportion (0.936 vs. 0.937, respectively) and the lower bound of the 97.5% CI exceeded 0.80 in each arm when disaggregated by time period during which loose pills vs. FDCs were offered. Although loose vs fixed pills did not seem to impact outcomes based on this analysis, it is certainly possible it could have with a larger population of patients. We hope any program considering rolling out 3HP would do so with the fixed-dose combination pills.
2. Did the authors consider also conducting analysis for all those who were assigned a strategy (DOT or SAT) compared to all those who were provided a choice (despite which strategy they chose) to assess whether just having a choice in their healthcare management improved their acceptance or completion?
   - **Response:** We had not considered this. We completed this analysis per the reviewer’s suggestion, however, given the high and similar acceptance/completion rates in the three arms, we are unable to show any difference in DOT + SAT vs. Choice arms: PR=1.01; 97.5% CI [0.98-1.04] p=0.416.
3. The article might benefit from a few more details in the methods section about how costs were collected and calculated.
   - **Response:** We have added in additional information about the costing methods in both the “Data collection” and “Statistical Analysis” sections of the Methods:
     i. *“Both study and clinic staff logged weekly time requirements for their duties, and clinical activities were observed using time-and-motion surveys. Time data were transferred to human resource cost based on the national average level of each type of staff’s hourly salary. Study staff completed monthly budgetary reviews of bills and receipts for resource-use data including overhead costs, study drugs, drug import fees, office supplies, internet charges, and lab consumables. Cost of transport reimbursement was calculated based on the total number of patients and their number of visits in each arm.”*
     ii. *“We estimated the total per patient cost of each delivery strategy from the health system perspective by compiling cost data from time and motion surveys, weekly time logs and monthly budgetary reviews.* *Costs were estimated in 2021 Ugandan Shillings and converted to US dollars using the average exchange rate in 2021 (1 USD=3,587.05 USh) (16).”*
4. Some information in the discussion re: formative work identifying barriers that were incorporated into the delivery strategies would be beneficial to describe in the context of the description of the strategies in the methods section.
   - **Response:** Thank you; while we report extensively on these methods and findings in our published trial protocol referenced in the manuscript, we agree that we can share some additional information in our discussion. Please see paragraph 3 of the Discussion for the edited section describing the formative work we completed to identify delivery strategy components: *“Our formative work identified three key barriers to 3HP completion: poor understanding of the need for preventive therapy, time and costs of accessing care, long wait times for medications, and difficulty incorporating a new medication into established routines. We addressed these barriers in all delivery strategies with counselling flipbooks designed to facilitate understanding of the importance of TPT for PLHIV, reimbursements covering transportation costs, streamlined clinic visits, and IVR phone call reminders (10). Survey data reflects that the barriers related to cost and transportation remained a challenge for some participants who were unsuccessful in completing 3HP. However, the overall high level of 3HP acceptance and completion with treatment integrated into routine care highlights the importance of identifying barriers through formative work and addressing those barriers during implementation.*

**Reviewer #5:** I considered the work of Prof. Adithya Cattamanchi and colleagues to be an extension of their published interim analysis, and it is a well-documented study. I would like the following explanations.

1. Lines 136-142: The paragraph's exclusion criteria seem repetitive because Supplement 1 already mentions them.
   - **Response:** We agree the two are redundant. However, other reviewers found these exclusion criteria to be important considerations and we therefore prefer not to minimize them by including only in the Supplement. We defer to the Editor whether to remove the exclusion criteria from the Results paragraph.
2. Was the relationship between education level and the informed Choice 3HP preventative treatment examined by the author?
   - **Response:** We had not examined this as the shared decision-making process was designed to be understandable and to include people without any formal education. However, based on the reviewer’s suggestion, we assessed if there was any difference in choice of SAT vs. DOT across educational strata, sex, and age within the choice arm. We found no evidence of differences in delivery strategy choice by education or age, but some differences across sex. We have added in the results of this analysis in Supplement 4 and the following sentence to the Results section: *“Choice of delivery strategy did not differ across educational strata or age; however, males were more likely than females to choose DOT (see* ***Supplement 4****).”*
3. All three treatment arms have similar median and range ages. How could that be?
   - **Response:** We thank the reviewer for their attention to detail and allowing us to double check the accurate reporting of age across study arms. Given that this was a randomized trial, we can expect that participant characteristics like age would be similar across study arms but agree the exact same medians and interquartile ranges in all three arms warrant further investigation. Using trial data that were provided with the full submission to PLOS Medicine, summaries can be generated demonstrating that all three arms indeed had a median age of 42, with an interquartile range of 36-48 (see detailed analytical output below). Had we asked participants to report their age to the day, then we anticipate that some differences might have emerged, but this finding partially reflects that we only asked participants to provide their age in years. The mean ages demonstrate slight differences in age across arms – but when rounded to the nearest year, even these means are the same (and the same as the median) as well. Generally, average ages reported here are similar to study populations among PLHIV taking 3HP in similar settings (e.g., the WHIP3TB trial).

```
bysort studyarm: sum age,d

-> studyarm = DOT
                            age
-------------------------------------------------------------
      Percentiles
Obs                 551
25%           36 			Sum of Wgt.         551

50%           42			Mean            42.0363
                			Std. Dev.      9.374363
75%           48


-> studyarm = SAT
                            age
-------------------------------------------------------------
      Percentiles
					Obs                 552
25%           36			Sum of Wgt.         552

50%           42			Mean           42.40399
                			Std. Dev.      9.126944
75%           48


-> studyarm = CHOICE
                             age
-------------------------------------------------------------
      Percentiles
    	Obs                 552
25%           36			Sum of Wgt.         552

50%           42			Mean           42.26812
                			Std. Dev.      9.404243
75%           48
```

4. Why were there more female participants in all three arms of the study, despite the fact that those who were pregnant, breastfeeding a baby, or planning to get pregnant within the next 120 days were not eligible?
   - **Response:** The 2020-2021 Uganda Population-based HIV Impact Assessment survey^1,2^ showed that the prevalence of HIV is higher among adult women (15 years and older) at 7.2% compared to men at 4.3%. In addition, recent studies on isoniazid preventive therapy among people living with HIV in Uganda showed that female participants ranged from 61% to 69%^3,4^. Finally, as of June 30th, 2023, the Mulago Immune Suppression Syndrome clinic (the trial site) located in Kampala, the capital city of Uganda, had a total of 16,548 clients in active care. Of these, 11,221 (68%) were women. Therefore, the predominance of women in this study was representative of the clinic's client population and observed national trends.
1. <https://phia.icap.columbia.edu/wp-content/uploads/2022/08/UPHIA-Summary-Sheet-2020.pdf>
2. <https://www.mediacentre.go.ug/media/release-preliminary-results-2020-uganda-population-based-hiv-impact-assessment>
3. Sensalire, S., et al., A prospective cohort study of outcomes for isoniazid prevention therapy: a nested study from a national QI collaborative in Uganda. AIDS Research and Therapy, 2020. 17(1): p. 28.
4. Amanya, I., et al., Isoniazid preventive therapy completion and factors associated with non-completion among patients on antiretroviral therapy at Kisenyi Health Centre IV, Kampala, Uganda. PLOS ONE, 2023. 18(8): p. e0277739.
5. What is prior Tuberculosis? Patients with active tuberculosis, suspicion of active tuberculosis, and those who previously completed treatment for tuberculosis were not eligible for the study.
   - **Response:** Thank you for the opportunity to clarify. You are correct that people with active tuberculosis, suspicion of active tuberculosis, and those who previously completed treatment for TB or TB infection **within the last 2 years** were not eligible for the study. However, people who completed treatment for TB or TB infection ≥2 years prior to the time of eligibility assessment were eligible for study participation. As shown in Table 1, 301 study participants (104 in the DOT arm, 108 in the SAT arm, and 89 in the Choice arm) completed treatment for TB or TB infection ≥2 years prior to study enrollment. We changed the row heading from “Prior TB” to “Prior treatment for tuberculosis (TB) or TB infection” in Table 1 for improved clarity and made similar changes in the Abstract and Methods sections.
