# Supplementary material for: Comparison of 3 optimized delivery strategies for completion of isoniazid-rifapentine (3HP) for tuberculosis prevention among people living with HIV in Uganda: A single-center randomized trial
Source: PLoS Med. 2024 Feb 20;21(2):e1004356. doi: 10.1371/journal.pmed.1004356 (PMC10914279; doi:10.1371/journal.pmed.1004356)
Supplement: Attachment — Submitted filename: Response to Reviewers_2nd review.docx [file pmed.1004356.s020.docx]

**Requests from Editors**

**ABSTRACT**

1. Abstract Background: Please provide the study question/hypothesis as the final sentence of the background section.
   - **Response:** Thank you for this suggestion; we have added in the following sentence from lines 82-85 of the revised manuscript: *“Our co-primary hypotheses were that high levels of acceptance and completion of 3HP could be achieved with delivery strategies optimized to overcome well-contextualized barriers and that 3HP acceptance and completion would be highest when PLHIV were provided an informed choice between delivery strategies.”*
2. Please remove the trial registration details from the Methods and Findings section and add it at the end of the abstract as a separate information.
   - **Response:** Trial registration details have been added as its own line at the end of the abstract as suggested (line 107).
3. In the last sentence of the Abstract Methods and Findings section, please clearly describe the main limitation(s) of the study's methodology. Editorial suggestion: The main limitation of the study is that it was conducted in a single center, and multicenter studies are now needed to confirm the feasibility and generalizability of facilitated 3HP delivery strategies in other settings.
   - **Response:** Thank you for this suggestion; we have added this limitation to the abstract (lines 101-102).

**AUTHOR SUMMARY**

1. Under ‘Why Was This Study Done?’, we feel the second bullet point describes differences to two previous implementation trials without clearly stating the reason for conducting the current study. Please revise. Editorial suggestion: Two previous implementation trials have evaluated the delivery of 12 weeks of isoniazid and rifapentine (3HP) through self-administered therapy (SAT) in high-burden settings and found variable treatment completion rates, in both studies, 3HP dosing and treatment supervision were provided by research staff rather than routine health workers.
   - **Response:** Thank you for the new suggested language, which we have incorporated into lines 136-139 in the “Author Summary” section of the revised manuscript.
2. Please revise the bullet points under ‘What Did the Researchers Do and Find?’. We feel that a bullet point describing treatment options is currently missing and that the interpretation of these findings should be limited to question 3 (What Do These Findings Mean?). Editorial suggestion: - In a pragmatic trial, 1,655 people living with HIV (PLHIV) at a high-volume clinic in Kampala, Uganda, received 3HP either by facilitated directly observed therapy (DOT) involving a pharmacy technician, facilitated self-administered therapy (SAT), or informed choice between facilitated DOT and facilitated SAT using a shared decision-making aid. - We found high levels of 3HP completion in the context of routine HIV/AIDS care. - The high completion rates were independent of therapy delivery strategy.
   - **Response:** We have included these edits in the updated “Author Summary” section of the manuscript and thank the editors for the suggested language.

**INTRODUCTION**

1. ll.134-135: Please provide reference for the sentence starting “Scaling up TPT is crucial...”.
   - **Response:** Two references have been added to the end of this sentence (line 156).
2. l.143: Please exchange ‘Both’ with ‘Two’.
   - **Response:** This revision has been made (line 165).

**METHODS AND RESULTS**

1. ll.209-226: We feel that the detailed descriptions of facilitated directly observed therapy (DOT), facilitated self-administered therapy, and informed choice delivery strategies provided in Supplement 2 should be included in the main manuscript. Please revise accordingly and remove Supplement 2.
   - **Response:** Detailed descriptions of trial study arms are now incorporated into the main body of the manuscript as suggested (pages 10-11, lines 230-261). As such, we have removed supplement 2 and renamed all attached supplements to the resubmission to be in accordance with this update.
2. ll.240-250: In the Methods section under “Outcomes”, we ask that you include a complete list of secondary outcomes (it is not necessary to include all sub-bullet points as listed in the published protocol), followed by a statement about which of them are reported in this paper. Please also mention that data on efficacy endpoints are still being collected.
   - **Response:** Thank you for this suggestion. Lines 281-295 (pasted also below) now include all secondary outcomes reported in this paper, and all that will be reported later. We also included the additional line about data on efficacy endpoints.

***“****Additional pre-specified secondary outcomes reported here include: adverse events leading to 3HP discontinuation, the fidelity of intervention and delivery strategy implementation (reimbursement metrics, time spent at clinical visits/time spent on 3HP-related activities, time spent completing the shared decision-making tool, proportion of IVR check-in calls, dosage reminders, and appointment reminders sent, proportion of participants who received follow-up/support actions following response to IVR check ins or missed appointments/doses, and proportion of doses confirmed using the 99DOTS platform), the acceptability of delivery strategies (median scores for domains within the patient satisfaction questionnaire regarding services received throughout the course or 3HP treatment, median scores for domains within the shared decision-making questionnaire regarding the shared decision-making process), and the per-patient cost of 3HP delivery from the health system perspective. Additional secondary outcomes that will be reported separately include cost-effectiveness, patient costs, thematic results from patient and healthcare provider in-depth and key informant interviews, self-reported patient barriers to TB preventive services, provider and clinic-level barriers to 3HP delivery, and data on active TB screening and TB incidence up to two years post-treatment (12). Data on efficacy endpoints are still being collected.”*

3. l.282: The terms gender and sex are not interchangeable (as discussed in https://urldefense.com/v3/__https://www.who.int/health-topics/gender__;!!LQC6Cpwp!r0PHIoBcpHru_4RKY82KwM2RT6R9djX4rkDWGle_LYH3JuLZg9xHwfh5K5ZB8AR9VnRJkGN_s3DlF41I4IiCgdWOng$ ); please use the appropriate term. We suggest using the word 'female' here.
   - **Response:** Thank you, this edit has been made.
4. I.286: Given that Supplement 4 contains 8 tables, we suggest specifying the reference (here: Table 1 in Supplement 4). Please revise throughout the main manuscript.
   - **Response:** These updates has been made throughout the main manuscript.
5. l.313: “one who died in a motor vehicle accident.” - We suggest that this statement be reworded to read "one who died for reasons unrelated to the trial". Since the study was conducted in a single center and at a specific time, we are concerned that this may expose the deceased individual.
   - **Response:** Thank you for the suggested replacement language – we have updated the manuscript to reflect the change (line 358).
6. Per CONSORT, please present adverse events in a table and discuss whether or not adverse events are thought to be related to treatment.
   - **Response:** Thank you, we have added in these details as a supplementary table (Supplement Table 1) which is attached to the resubmission and referenced in the manuscript on line 398.

**DISCUSSION**

1. l.424: “Informed choice was introduced as a novel, person-centered…” – please remove the word ‘novel’.
   - **Response:** We have removed this word per the suggestion.
2. l.446: Please remove the word “extremely”.
   - **Response:** We have removed this word per the suggestion.

**FIGURES**

1. Figure 1: Please define '3HP'. Please note that it is not necessary to define abbreviations within the figure and additionally list these abbreviations below the figure, either one is sufficient.
   - **Response:** Thank you, we have added in the following definition of 3HP as a footnote to Figure 1: *“3HP=once-weekly isoniazid and rifapentine for 12 weeks”.* We have removed the redundant abbreviations that were included in both the figure and below the figure.
2. Figure 1: In the boxes directly above the numbers analyzed: In the SAT group, it is not necessary to indicate that "0 never initiated therapy", please remove.
   - **Response:** Thank you, we have updated figure 1 to remove the phrase “0 never initiated therapy” as suggested.
3. Figure 4: We suggest adding footnotes detailing what the 99DOTS platform is and what human resources includes.
   - **Response:** We thank the editors for these suggestions. We have added two footnotes to the bottom of Figure 4 to include a description of the 99DOTS platform and an explanation for what was included as “Human Resources” costs.

**REFERENCES**

1. When specifying the date of access, please write “accessed” instead of “cited”.
   - **Response:** Thank you, these corrections have been made to the references section.

**SUPPLEMENTARY MATERIAL**

1. Please be sure to define 3HP where applicable.
   - Response: We have added a footnote defining 3HP as “twelve weeks of once-weekly isoniazid and rifapentine” to the following supplementary materials:
     i. Supplement 1
     ii. Supplement 3: Tables 2, 4, 5, 6 and 7
     iii. Supplement Figure 3
     iv. Supplement Figure 4
     v. Supplement Table 1
2. Please be sure to define what the strategy “Choice” encompasses where applicable.
   - **Response:** Thank you, we have updated footnotes to the following supplementary materials to include a definition of “Choice”:
     i. Supplement 3: Tables 1, 4, 8
3. Supplement Figure 1: Please also define the meaning of the yellow boxes.
   - **Response:** We have edited the caption for the figure included in Supplement Figure 1 to now include a definition of the yellow boxes, also pasted below:

***“…****and yellow squares indicate doses recorded via toll-free phone call that were reported using a phone number shared by another participant in the study (SAT only).”*

4. Supplement Figure 3: Please change ‘women’ and ‘men’ to ‘female’ and ‘male’.
   - **Response:** Thank you, this update has been made.
5. Supplement Figure 4: Please change ‘women’ and ‘men’ to ‘female’ and ‘male’. Please mention in the figure description that the colours indicate the pre-specified subgroups (red = sex, blue = age, purple = time on ART, green = prior TB status).
   - **Response:** Thank you, these updates have been made.

**SOCIAL MEDIA**

1. To help us extend the reach of your research, please provide any X (formerly known as Twitter) handle(s) that would be appropriate to tag, including your own, your coauthors’, your institution, funder, or lab. Please respond to this email with any handles you wish to be included when we tweet this paper.
   - **Response:** Please see below for Twitter handles to tag:
     i. Adithya Cattamanchi: @acattamanchi
     ii. David Dowdy: @davidwdowdy
     iii. Fred Semitala: @SemitalaFred
     iv. Margaret Handley: @MAHandley
     v. UCSF PRISE Center: @ImSatUCSF
     vi. UCSF Center for TB: @UCSF_CTB

**Comments from Reviewers:**

1. Reviewer #1: We thank the authors for addressing our previous concerns.
   - **Response:** Thank you for your review.
2. Reviewer #2: The authors have been mostly responsive to the prior comments, and the manuscript is suitable for publication. However, given the dis-aggregated adherence measures were similar across 3 study arms, and the aggregated results have been previously published, this manuscript adds little value to the prior publication.  The addition of the secondary outcomes was helpful, but also did not add much value to this manuscript.
   - **Response:** Thank you for the additional review of the revised manuscript and for the suggestions for improvement for the *PLOS Medicine* readership.
3. Reviewer #3: The revisions and author responses have addressed all of my previous comments and concerns about this manuscript. The revised version is clearly written and improved by the incorporation of suggestions made by my fellow reviewers. In the Methods and Findings section of the abstract, consider the following very minor change for improved readability 'facilitated SAT using a shared decision-making aid' would perhaps be clearer as 'facilitated SAT using a shared decision-making aid (Choice)' to be consistent with the designation used later in the manuscript for the third group of participants.
   - **Response:** Thank you for the suggested edit, which we have included in the revised manuscript.
4. Reviewer #4: I appreciate the thoughtful and comprehensive response to the reviewers' comments. All pending queries have been resolved and I support this manuscript for publication.
   - **Response:** Thank you for the support of our manuscript for publication.
